# Enantioselective total synthesis of putative dihydrorosefuran, a monoterpene with an unique 2,5-dihydrofuran structure

**DOI:** 10.3762/bjoc.18.132

**Published:** 2022-09-19

**Authors:** Irene Torres-García, Josefa L López-Martínez, Rocío López-Domene, Manuel Muñoz-Dorado, Ignacio Rodríguez-García, Miriam Álvarez-Corral

**Affiliations:** 1 Departamento Química Orgánica, Universidad de Almería, ceiA3. E04120 Almería, Spain

**Keywords:** Ag(I) cyclization, allenylation, CpTiCl_2_, 2,5-dihydrofurans, monoterpenes

## Abstract

An original synthesis of the structure of dihydrorosefuran, a compound allegedly identified in *Artemisia pallens* and *Tagetes mendocina*, has been developed. The key steps in the five-step 36% overall yield synthesis are a CpTi^III^Cl_2_ mediated Barbier-type allenylation of a linear aldehyde and the formation of a 2,5-dihydrofuran scaffold through a Ag(I)-mediated cyclization. Neither of the reported spectral data for dihydrorosefuran match those of the synthetic product, suggesting that the isolated compound from *Tagetes mendocina* is in fact the natural product rosiridol, while the real structure of the product from *Artemisia pallens* remains unknown.

## Introduction

*Artemisia pallens* is an aromatic plant from southern India whose essential oil, known as Davana oil, has shown increasing interest mainly for its use in some beverages, cakes, pastries, etc., as well as in the perfumery industry [1]. In addition, *A. pallens* has been used in Indian traditional medicine (Ayurveda) for the treatment of measles, cough, cold, depression, diabetes, and high blood pressure [2]. More recently other biological activities have been reported, such as the blood glucose lowering effect of *A. pallens* [3–4], and its anti-asthmatic potential [5].

**Scheme 1:**
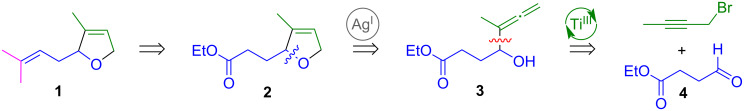
Retrosynthetic scheme of the target molecule **1**.

A component responsible for the fresh and floral odor of the essential oil was isolated from Davana oil and was assigned as a 2,5-dihydrofuranic monoterpenoid (compound **1** in Scheme 1) and named dihydrorosefuran [6–8]. Furthermore, the same structure was attributed to an isolated substance from the Argentinean herb *Tagetes mendocina* [9], although not all of its spectroscopic features did match point by point with those previously reported. This made us think that this could be a case of misassigned natural product [10], hence we decided to perform its total synthesis.

## Results and Discussion

Our synthetic strategy is based on two metal-mediated steps (Scheme 1). In this way, we thought that the 2,5-dihydrofuran structural motif that is found in the target molecule **1** could be prepared through a Ag(I)-induced intramolecular addition of the hydroxy group to the terminal double bond of the allene in compound **3**. Another key step is the Ti(III)-mediated straightforward synthesis of this α-hydroxyallene, which could be achieved through a regioselective Barbier*-*type coupling of a propargylic halide (1-bromo-2-butyne) with the aldehyde **4** mediated by the organometallic half-sandwich complex [CpTi^III^Cl_2_] [11–12].

**Scheme 2:**
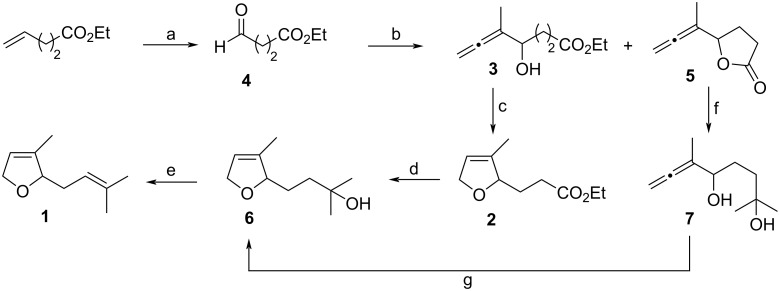
Synthesis of dihydrofuran-monoterpenoid **1**. a) i. O_3_, −78 °C; ii. PPh_3_, rt, 76%; b) 1-bromobut-2-yne, CpTiCl_3_, Mn and THF (**3**: 71–81%, **5**: 0–10%); c) AgNO_3_, Me_2_CO, 88%; d) MeMgBr, Et_2_O, 90%; e) Amberlyst^®^-15, DCM, 74%; f) MeMgBr, Et_2_O, 83%; g) AgNO_3_, Me_2_CO, 75%.

Following this retrosynthetic proposal, our route starts from ethyl 4-oxobutanoate (**4**) [13] which was prepared by ozonolysis of commercially available ethyl pent-4-enoate (Scheme 2). Coupling of the aldehyde **4** with 1-bromobut-2-yne in the presence of CpTi^III^Cl_2_ (generated in situ by reduction of CpTiCl_3_ with Mn) afforded α-hydroxyallene **3**. We have recently described that this Barbier-type reaction affords α-hydroxyallenes as major products, mixed with smaller amounts of homopropargylic alcohols, either if the reaction is performed with stoichiometric amounts of CpTiCl_3_ or if catalytic amounts are used [12]. However, using the particular substrates in this approach, the allenic compound **3** was exclusively formed, in a satisfactory 81% yield [14]. It is also important to control the pH during the reaction workup, as some contamination of the product with lactone **5** can arise at low pH values, which goes in detriment of the yield. The 2,5-dihydrofuran ring in target compound **1** was obtained through a Ag(I)-mediated intramolecular addition of the hydroxy to the allene group, a process that transformed allene **3** into compound **2**. The isopropenyl residue of the target compound **1** was assembled through a two-step sequence. The first one was the addition of an excess of methylmagnesium bromide to the ester **2**, that completed the carbon skeleton. The second step was the pH-controlled regioselective dehydration of the tertiary alcohol **6** with amberlyst-15^®^ leading to the monoterpene **1**. Other systems tested for the elimination of the hydroxy group in **6** were pyridinium *p*-toluenesulfonate (PPTS) and camphorsulfonic acid (CSA), that gave poorer results, failing to afford a single product. On the other hand, lactone **5** could also be transformed into alcohol **6** through a simple change in the order of the reactions: addition of methylmagnesium bromide to **5** afforded **7**, which was then transformed into **6** by the Ag(I)-mediated cyclization (Scheme 2).

**Scheme 3:**
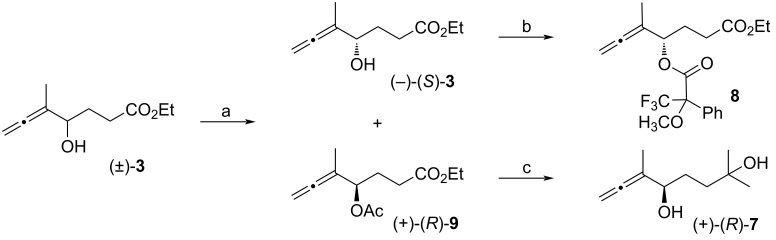
Racemic resolution of allenol **3** and synthesis of derivatives. a) Lipase AK, vinyl acetate, *t*-BuOMe, 30 °C, ((−)-(*S*)-**3**: 46%, 90% ee, (+)-(*R*)-**9**: 39%, 95% ee); b) *N*,*N*’-dicyclohexylcarbodiimide (DCC), dimethylaminopyridine (DMAP), (*S*) or (*R*)-(−)-α-methoxy-α-(trifluoromethyl)phenylacetic acid, 66% [17], c) MeMgBr (5 equiv), Et_2_O, 67%.

Once we had synthesized racemic compound **1**, we designed a chiral version using a stereoselective kinetic resolution of allenol **3** via lipase AK-catalyzed acetylation [15]. In this way, unaltered, (−)-hydroxyallene **3** could be separated from (+)-acetyl derivative **9** through standard column chromatography (Scheme 3). Enantiomeric excesses of (−)-**3** and (+)-**9** were determined by chiral HPLC analyses. Analysis of the NMR data of the Mosher's derivatives of **8** suggested (*S*) configuration for the alcohol (−)-**3** [16].

On the other hand, enantiopure acetate (+)-(*R*)-**9** was transformed into diol (+)-(*R*)-**7** by the addition of an excess of MeMgBr. Finally, these enantiopure compounds, α-hydroxyallene (−)-(*S*)-**3** and diol (+)-(*R*)-**7**, can be used to prepare both enantiomers of compound **1** following the procedures shown in Scheme 2.

Unfortunately, once the racemic synthesis was successfully completed and the chiral design was fulfilled, it was found that the spectroscopic data of compound **1** did not match neither with those published for the allegedly dihydrorosefuran isolated from *Artemisia pallens* nor with those reported for the compound from *Tagetes mendocina* (see Table 1 and Table 2). The ^13^C NMR data of compound **1** are quite similar to those of the natural product isolated from *T. mendocina,* except for the signals of the oxygenated carbons (C2 and C5). The same behavior pattern can be observed in the ^1^H NMR data. This made us think that the natural product of *T. mendocina* could have an acyclic skeleton instead of a dihydrofuran one. For this reason, we propose this compound should be the diol called rosiridol (Table 1), a substance that has been isolated from other natural sources [18–19], whose structure was also confirmed by total synthesis some years ago [20]. Comparison of NMR data (Table 1) confirmed the initial suspicion. We are still intrigued about the real structure of the natural product isolated from *A. pallens*. However, it must be considered that this product was elucidated using low frequency NMR machines, which suggests that further research on the chemical composition of this oil is needed.

**Table 1:** ^1^H NMR data of isolated and synthetic products.^a^

	sources of claimed dihydrorosefuran		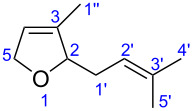 **1**	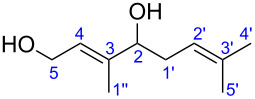 rosiridol^b^
	*A. pallens*^a^*^,^*^c^ [7–8]	*T. mendocina*^d^ [9]	this work synthesis^e^	literature synthesis^d^ [20]

H2	4.04 (td, *J* = 8.0, 1.0 Hz)	4.03 (t, *J* = 8 Hz)	4.67 (br s)	4.02–3.99 (m)
H4	5.07 (td, *J* = 8.0, 1.0 Hz)	5.66 (t, *J* = 8 Hz)	5.51 (br s)	5.67–5.62 (m)
H5	4.55 (d, *J* = 7.0 Hz)	4.21 (dd)	4.62–4.53 (m)	4.24–4.15 (m)
H1'	2.30 (m)	2.24 (m)	2.41 (m), 2.20 (ddd, *J* = 14.0, 7.0, 6.5 Hz)	2.28–2.19 (m)
H2'	5.32 (t, *J* = 7.0 Hz)	5.13 (t, *J* = 8 Hz)	5.21 (t, hept, *J* = 6.9, 1.5 Hz)	5.13–5.09 (m)
H4'	1.67 (s)	1.75 (s)	1.74 (s)	1.73 (d, *J* = 1.1 Hz)
H5'	1.60 (s)	1.66 (s)	1.66 (s)	1.64 (s)
H1"	2.05 (s)	1.69 (s)	1.72 (s)	1.67 (s)

^a^CDCl_3_ in all cases; ^b^arbitrary numbering for comparison purposes; ^c^80 MHz; ^d^400 MHz; ^e^500 MHz.

**Table 2:** ^13^C NMR data of isolated and synthetic products^a^.

	sources of claimed dihydrorosefuran		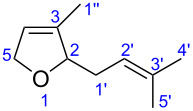 **1**	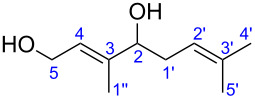 rosiridol^b^
	*A. pallens*^a,c^ [7–8]	*T. mendocina*^d^ [9]	this work synthesis^e^	literature synthesis^d^ [20]

C2	64.0	76.4	87.5	76.4
C3	145.5	140.4	138.1	140.2
C4	129.3	124.5	120.7	124.5
C5	61.0	59.1	74.5	59.0
C1'	39.8	34.2	32.9	34.1
C2’	118.0	119.7	119.7	119.8
C3'	142.0	135.3	133.6	135.0
C4'	26.2	25.9	25.9	25.8
C5'	24.8	18.0	18.0	18.0
C1"	17.5	12.2	12.5	12.0

^a^CDCl_3_ in all cases; ^b^arbitrary numbering for comparison purposes; ^c^80 MHz; ^d^100 MHz; ^e^125 MHz.

## Conclusion

In summary, we have proved that the two-step sequence Ti^III^ allenylation–Ag^I^ cyclization is a simple and efficient strategy for the preparation of the 2,5-dihydrofuran moiety present in many natural products. In fact, we have achieved the total synthesis of the 2,5-dihydrofuran structure **1**. After systematic data analysis of our prepared compound and those in the literature, it can be concluded that the proposed structure of the product isolated from *Artemisia pallens* oil, dihydrorosefuran, is not correct. In addition, it is clear that the compound isolated from *Tagetes mendocina* is the acyclic diol named rosiridol.

## Experimental

### Ti-induced allenylation of ethyl 4-oxobutanoate (**4**)

Under an Ar atmosphere, dry THF (8 mL) that was deoxygenated prior to use was added to a mixture of CpTiCl_3_ (329 mg, 1.50 mmol) and Mn dust (165 mg, 3.00 mmol) resulting in a green suspension. Then, a solution of ethyl 4-oxobutanoate (**4**, 196 mg, 1.50 mmol) and 1-bromobut-2-yne (0.27 mL, 3.00 mmol) in THF (2 mL) was dripped and the mixture was stirred for 2.5 hours. The mixture was filtered, diluted with EtOAc, washed with 3% HCl and brine, and dried (anhydrous MgSO_4_), and the solvent was removed. The residue was purified by flash chromatography (*n-*hexane/EtOAc 8:2) to afford ethyl 4-hydroxy-5-methylhepta-5,6-dienoate (**3**, 225 mg, 81%) isolated as light yellow oil. IR (ATR) *ν* (cm^−1^): 3434, 2972, 2928, 1958, 1723, 1436, 1374, 1172, 1028, 925, 853; ^1^H NMR (300 MHz, CDCl_3_) δ 4.77 (dq, *J* = 2.3, 3.2 Hz, 2H), 4.12 (q, *J* = 7.1 Hz, 2H), 4.05 (m, 1H), 2.42 (t, *J* = 7.2 Hz, 2H), 2.18 (s, 1H), 2.04–1.77 (m, 2H), 1.70 (td, *J* = 0.5, 3.2 Hz, 3H), 1.25 (t, *J* = 7.1 Hz, 3H) ppm; ^13^C NMR (75 MHz, CDCl_3_, DEPT) δ 204.8 (C), 174.0 (C), 101.6 (C), 77.0 (CH_2_), 71.5 (CH), 60.5 (CH_2_), 30.4 (CH_2_), 30.0 (CH_2_), 14.5 (CH_3_), 14.2 (CH_3_) ppm; HRMS–ESI (Q-TOF, *m/z*): [M + H]^+^ calcd for C_10_H_17_O_3_, 185.1178; found, 185.1158. A lactone is formed as side product (0–10%) when the HCl solution used for the workup has a concentration higher than 3%. Compound **5**: IR (ATR) *ν* (cm^−1^): 2982, 2927, 1960, 1772, 1427, 1331, 1162, 974, 918, 855; ^1^H NMR (300 MHz, CDCl_3_) δ 4.89 (m, 1H), 4.86 (m, 2H), 2.55 (m, 2H), 2.30 (m, 2H), 1.79 (t, *J* = 3.1 Hz, 3H) ppm; ^13^C{^1^H} NMR (75 MHz, CDCl_3_, DEPT) δ 206.0 (C), 177.0 (C), 98.0 (C), 80.4 (CH), 77.5 (CH_2_), 28.5 (CH_2_), 26.1 (CH_2_), 15.0 (CH_3_) ppm; HRMS–ESI (Q-TOF, *m/z*): [M + H]^+^ calcd for C_8_H_11_O_2_,139.0759; found, 139.0782.

### Silver(I)-promoted cyclization of ethyl 4-hydroxy-5-methylhepta-5,6-dienoate (**3**)

A solution of the allenol **3** (65 mg, 0.35 mmol) in acetone (2 mL) was added to a suspension of AgNO_3_ (120 mg, 0.70 mmol) in acetone (1.5 mL) in the absence of light, and the mixture was stirred at 40 °C overnight. Brine was added and the mixture was extracted with Et_2_O. The organic phase was dried over anhydrous MgSO_4_, and concentrated under reduced pressure. The residue was purified by silica gel flash column chromatography (*n-*hexane/EtOAc 9:1) to afford ethyl 3-(3-methyl-2,5-dihydrofuran-2-yl)propanoate (**2**, 57 mg, 88%) isolated as colorless oil. IR (ATR) *ν* (cm^−1^): 2969, 2927, 2849, 1731, 1442, 1376, 1251, 1160, 1092, 1026, 895, 734. ^1^H NMR (300 MHz, CDCl_3_) δ 5.53 (m, 1H), 4.69 (br s, 1H), 4.56 (m, 2H), 4.15 (q, *J* = 7.1 Hz, 2H), 2.39 (m, 2H), 2.12–2.06 (m, 1H), 1.83–1.74 (m, 1H), 1.72 (br s, 3H), 1.27 (t, *J* = 7.1 Hz, 3H) ppm; ^13^C{^1^H} NMR (75 MHz, CDCl_3_, DEPT) δ 173.9 (C), 137.2 (C), 121.3 (CH), 86.4 (CH), 74.7 (CH_2_), 60.3 (CH_2_), 29.4 (CH_2_), 28.9 (CH_2_), 14.2 (CH_3_), 12.3 (CH_3_) ppm; HRMS–ESI (Q-TOF, *m/z*): [M + H]^+^ calcd for C_10_H_17_O_3_,185.1172; found, 184.1162.

### Synthesis of 2-methyl-4-(3-methyl-2,5-dihydrofuran-2-yl)butan-2-ol (**6**)

Under an N_2_ atmosphere, methylmagnesium bromide (3 M in Et_2_O, 0.075 mL, 0.23 mmol) was diluted with dry Et_2_O (1.5 mL). A solution of ethyl 3-(3-methyl-2,5-dihydrofuran-2-yl)propanoate (**2**, 16 mg, 0.087 mmol) in Et_2_O (1 mL) was added dropwise and the reaction mixture was stirred for 3 hours at room temperature. The reaction was quenched with saturated NH_4_Cl and extracted with EtOAc. The combined organic layer was washed with saturated NaHCO_3_, brine, and dried over anhydrous MgSO_4_. The solvent was evaporated in vacuum and the residue was purified using column chromatography (*n-*hexane/EtOAc 7:3) to give 2-methyl-4-(3-methyl-2,5-dihydrofuran-2-yl)butan-2-ol (**6**, 169 mg, 90%) isolated as colorless oil. IR (ATR) *ν* (cm^−1^): 3425, 2969, 2922, 2852, 1636, 1444, 1382, 1089, 1057, 1025; ^1^H NMR (300 MHz, CDCl_3_) δ 5.51 (br s, 1H), 4.68 (br s, 1H), 4.58 (m, 2H), 2.05 (br s, 1H), 1.82 (m, 1H), 1.72 (br s, 3H), 1.57 (m, 3H), 1.24 (s, 6H) ppm; ^13^C{^1^H} NMR (75 MHz, CDCl_3_, DEPT) δ 137.8 (C), 120.7 (CH), 87.7 (CH), 74.5 (CH_2_), 70.4 (C), 38.5 (CH_2_), 29.5 (CH_3_), 29.4 (CH_3_), 28.3 (CH_2_), 12.5 (CH_3_) ppm; HRMS–ESI (Q-TOF, *m/z*): [M + H]^+^ calcd for C_10_H_19_O_2_, 171.1385; found, 171.1368.

### Synthesis of 3-methyl-2-(3-methylbut-2-en-1-yl)-2,5-dihydrofuran (**1**)

The reaction of amberlyst^®^-15 (dry, 97 mg) and 2-methyl-4-(3-methyl-2,5-dihydrofuran-2-yl)butan-2-ol (**6**, 97 mg, 0.57 mmol), based on the previously reported literature procedure [21], afforded 3-methyl-2-(3-methylbut-2-en-1-yl)-2,5-dihydrofuran (**1**, 64 mg, 74%) isolated as colorless oil. IR (ATR) *ν* (cm^−1^): 3066, 2965, 2918, 2843, 1668, 1445, 1377, 1080, 975, 850, 778; ^1^H NMR (500 MHz, CDCl_3_) δ 5.51 (br s, 1H, H4), 5.21 (t, hept, *J* = 7.0, 1.5 Hz, 1H, H2'), 4.67 (br s, 1H, H2), 4.62–4.53 (m, 2H, H5), 2.41 (m, 1H, H1'a), 2.20 (ddd, *J* = 14.0, 7.0, 6.5 Hz, 1H, H1'b), 1.74 (d, *J* = 1.5 Hz, 3H, H4'*), 1.72 (m, 3H, H1''), 1.66 (br s, 3H, H5'*) ppm; ^13^C{^1^H} NMR (125 MHz, CDCl_3_, DEPT) δ 138.1 (C, C3), 133.6 (C, C3'), 120.7 (CH, C4), 119.7 (CH, C2'), 87.5 (CH, C2), 74.5 (CH_2_, C5), 32.9 (CH_2_, C1'), 25.9 (CH_3_, C4'*), 18.0 (CH_3_, C5'*), 12.5 (CH_3_, C1'') ppm (*may be interchanged); HRMS–ESI (Q-TOF, *m/z*): [M + H]^+^ calcd for C_10_H_17_O, 153.1274; found, 153.1262.

## Supporting Information

File 1 Experimental procedures, characterization of other substances, and copies of IR, NMR spectra and HPLC chromatograms.
