# Supplementary material for: Enantioselective total synthesis of putative dihydrorosefuran, a monoterpene with an unique 2,5-dihydrofuran structure
Source: Beilstein J Org Chem. 2022 Sep 19;18:1264–9. doi: 10.3762/bjoc.18.132 (PMC9520846; doi:10.3762/bjoc.18.132)
Supplement: File 1 — Experimental procedures, characterization of other substances, and copies of IR, NMR spectra and HPLC chromatograms. [file Beilstein_J_Org_Chem-18-1264-s001.pdf]

BEILSTEIN JOURNAL OF ORGANIC CHEMISTRY

Supporting Information

for

Enantioselective total synthesis of putative dihydrorosefuran, a monoterpene with an unique 2,5-dihydrofuran structure

Irene Torres-García, Josefa L. López-Martínez, Rocío López-Domene, Manuel Muñoz-Dorado, Ignacio Rodríguez-García and Miriam Álvarez-Corral

Beilstein J. Org. Chem. **2022,** *18,* 1264–1269. doi:10.3762/bjoc.18.132

Experimental procedures, characterization of other substances, and copies of IR, NMR spectra and HPLC chromatograms

License and Terms: This is a supporting information file under the terms of the Creative Commons Attribution License (https://creativecommons.org/licenses/by/4.0). Please note that the reuse, redistribution and reproduction in particular requires that the author(s) and source are credited and that individual graphics may be subject to special legal provisions.
The license is subject to the *Beilstein Journal of Organic Chemistry* terms and conditions: (https://www.beilstein-journals.org/bjoc/terms)

Table of contents

Experimental section S2
General remarks ... S2
Synthesis of ethyl 4-oxobutanoate (**4**) ... S3
Synthesis of 2,6-dimethylocta-6,7-diene-2,5-diol (**7**) ... S3
Silver (I) promoted cyclization of 2,6-dimethylocta-6,7-diene-2,5-diol (**7**) ... S3
Enzymatic kinetic resolution of α-allenic alcohols ... S4
Synthesis of (*R*)-2,6-dimethylocta-6,7-diene-2,5-diol ((+)-(*R*)-**7**) ... S4
Synthesis of the Mosher's derivatives of compound (−)-(*S*)-**3** ... S5
Synthesis of the Mosher's derivative of racemic compound (±)-**3** ... S6
References ... S6

NMR and IR spectra ... S7
^{1}H NMR, DEPT 135, ^{13}C NMR, HSQC, HMBC and IR of 3-methyl-2-(3-methylbut-2-en-1-yl)-2,5-dihydrofuran (**1**) ... S7
^{1}H NMR, DEPT 135, ^{13}C NMR and IR of ethyl 3-(3-methyl-2,5-dihydrofuran-2-yl)propanoate (**2**) . S12
^{1}H NMR, DEPT 135, ^{13}C NMR and IR of ethyl 4-hydroxy-5-methylhepta-5,6-dienoate (**3**) ... S15
^{1}H NMR, DEPT 135, ^{13}C NMR and IR of ethyl 4-oxobutanoate (**4**) ... S18
^{1}H NMR, DEPT 135, ^{13}C NMR and IR of 5-(buta-2,3-dien-2-yl)dihydrofuran-2(3*H*)-one (**5**) ... S21
^{1}H NMR, DEPT 135, ^{13}C NMR and IR of 2-methyl-4-(3-methyl-2,5-dihydrofuran-2-yl)butan-2-ol (**6**) ... S24
^{1}H NMR, DEPT 135, ^{13}C NMR and IR of 2,6-dimethylocta-6,7-diene-2,5-diol (**7**) ... S27
^{1}H NMR, DEPT 135 and ^{13}C NMR of ethyl (*S*)-5-methyl-4-(((*S*)-3,3,3-trifluoro-2-methoxy-2-phenylpropanoyl)oxy)hepta-5,6-dienoate ((*4S,2´S*)-**8**) ... S30
^{1}H NMR, DEPT 135 and ^{13}C NMR of ethyl (*S*)-5-methyl-4-(((*R*)-3,3,3-trifluoro-2-methoxy-2-phenylpropanoyl)oxy)hepta-5,6-dienoate ((*4S,2´R*)-**8**) ... S32
^{1}H NMR analysis of diastereomeric MTPA esters ... S34
^{19}F NMR: a) (*4S,2´S*)-8; b) (*4S,2´R*)-**8** ... S34
^{1}H NMR of diastereomeric mixture (*4S,2´S*)-8 and (*4R,2´S*)-**8** ... S35
^{1}H NMR, DEPT 135, ^{13}C NMR and IR of (*R*)-ethyl 4-acetoxy-5-methylhepta-5,6-dienoate ((+)-**9**) S36
HPLC data ... S39

Experimental section

General remarks

NMR spectra were recorded on Bruker Nanobay Avance III HD 300 MHz, Avance III HD 500 MHz and Avance III HD 600 MHz spectrometers. Proton-decoupled ^{13}C NMR spectra and DEPT-135 were measured in all cases. When required, HSQC and HMBC experiments were used for signal assignation. Chemical shifts (δ) are expressed in ppm and coupling constants (*J*) in hertz (Hz). Chemical shifts are reported using $CDCl_3$ as internal reference. IR Spectra were recorded with a Bruker Alpha spectrometer. Mass spectra were recorded in a Waters Xevo by LC-QTof-MS by electrospray ionization. The samples were analyzed by high performance liquid chromatography (HPLC) using an Agilent 1100 quaternary pump. The chromatographic columns used were Daicel Chiracel OD-H and Daicel Chiralpack IA. An isocratic elution of a mixture of hexane/isopronaol is all that is needed to analyze the compounds, and filtration is the only sample preparation required before injection. Separations are performed at a temperature of 25 °C and at flow rates of 0.4–0.5 mL/min. A UV detector with a diode array was also used and the compounds of interest were quantified at wavelengths 210 nm. All reactions were monitored by thin-layer chromatography (TLC) carried out on 0.2 mm DC-Fertigfolien Alugram® XtraSil G/UV254 silica gel plates. The TLC plates were visualized with UV light and 7% phosphomolybdic acid or $KMnO_4$ in water/heat. Flash chromatography was performed on silicagel 60 (0.04–0.06 mm). Commercially available chemicals were obtained from Aldrich Chemical Co., Acros, Alfa Aesar, and TCI and were used as received. In all experiments involving Ti(III), reactions were performed under argon atmosphere, using oven-dried glassware in all cases. THF was distilled from Na/benzophenone under argon, and was deoxygenated prior to use.

Synthesis of ethyl 4-oxobutanoate (4)

Ozone was bubbled through a solution of ethyl pent-4-enoate (1.22 g, 9.52 mmol) in DCM (20 mL) at −78 ºC until the solution turned blue. Then, PPh_3 (3 g, 11.42 mmol) was added and the mixture was stirred overnight at room temperature. The solvent was removed in vacuum and the residue purified by silica gel flash column chromatography (pentane/diethyl ether 7:3) to afford ethyl 4-oxobutanoate (**4**, 935 mg, 76%) as colorless oil. ^{1}H NMR and IR spectral data are in agreement with literature values [1]. ^{13}C{^{1}H}NMR (75 MHz, $CDCl_3$, DEPT) δ (ppm) 200.1 (CH), 172.3 (C), 60.8 (CH_2), 38.6 (CH_2), 26.6 (CH_2), 14.2 (CH_3).

Synthesis of 2,6-dimethylocta-6,7-diene-2,5-diol (7)

To a solution of methylmagnesium bromide (3 M in Et_2O, 0.14 mL, 0.43 mmol) in anhydrous Et_2O (1 mL), a solution of 5-(buta-2,3-dien-2-yl)-dihydrofuran-2(3*H*)-one (**5**, 24 mg, 0.17 mmol) in anhydrous diethyl ether (0.6 mL) was slowly added. The mixture was stirred under N_2 at room temperature for 40 min. The reaction was quenched with saturated NH_4Cl and extracted with ethyl acetate. The combined organic layer was washed with saturated $NaHCO_3$ and brine, dried over anhydrous $MgSO_4$. The solvent was removed in vacuum to give **7** (24 mg, 83%) as colorless oil. IR (ATR) ν (cm^{-1}) 3376, 2970, 2928, 2872, 1959, 1646, 1377, 1262, 1213, 1152, 1058, 1024, 907, 846, 803. ^{1}H NMR (300 MHz, $CDCl_3$) δ (ppm) 4.80 (dq, J = 5.4, 3.0 Hz, 2H), 4.07 (m, 1H), 2.08 (br s, 2H), 1.73 (t, J = 3.0 Hz, 3H), 1.64 (m, 4H), 1.25 (s, 6H).^{13}C{^{1}H} NMR (75 MHz, $CDCl_3$, DEPT) δ (ppm) 204.9 (C), 102.0 (C), 76.8 (CH_2), 72.7 (CH), 70.7 (C), 39.4 (CH_2), 29.8 (CH_2), 29.5 (CH_3), 29.3 (CH_3), 14.5 (CH_3). HRMS (ESI/Q-TOF) *m/z*: $[M+H]^+$ calcd for $C_{10}H_{19}O_2$ 171.1385; found 171.1397.

Silver(I)-promoted cyclization of 2,6-dimethylocta-6,7-diene-2,5-diol (7)

A solution of the allenol **7** (16 mg, 0.09 mmol) in acetone (1.5 mL) was added to a suspension of $AgNO_3$ (30 mg, 0.19 mmol) in acetone (1.5 mL) in the absence of light, and the mixture was stirred at 40 °C overnight. Brine was added and the mixture was extracted with Et_2O. The organic phase was dried over anhydrous $MgSO_4$, and concentrated under reduced pressure to

afford 2-methyl-4-(3-methyl-2,5-dihydrofuran-2-yl)butan-2-ol (**6**, 12 mg, 75%) that was isolated as colorless oil.

Enzymatic kinetic resolution of α-allenic alcohols

Based on the previously literature procedure [2], the reaction of ethyl 4-hydroxy-5-methylhepta-5,6-dienoate (**3**, 0.13 g, 0.71 mmol), lipase AK (35.5 mg, 20,000 U/g) and vinyl acetate (0.53 mL, 5.68 mmol) in methyl *tert*-butyl ether (7 mL), after purification by flash chromatography (*n*-hexane/Et_2O 7:3), provided the desired compounds including ethyl (*S*)-4-hydroxy-5-methylhepta-5,6-dienoate ((−)-(*S*)-**3**, 60 mg, 46%, 90% ee), $[\alpha]^{25}_{D}$ -9.2 (*c* 0.037, $CHCl_3$), and ethyl (*R*)-4-acetoxy-5-methylhepta-5,6-dienoate ((+)-(*R*)-**9**, 63 mg, 39%, 95% ee), $[\alpha]^{25}_{D}$ +115 (*c* 0.042, $CHCl_3$), as light yellow oils. Enantiomeric excess (ee) was determined by chiral HPLC (see HPLC Data). Compound (+)-(*R*)-**9**: IR (ATR) ν (cm^{-1}) 2982, 2937, 1961, 1731, 1431, 1371, 1227, 1178, 1020, 854. ^{1}H NMR (300 MHz, $CDCl_3$) δ (ppm) 5.23 (t, *J* = 6.5 Hz, 1H), 4.78 (m, 2H), 4.15 (q, *J* = 7.1 Hz, 2H), 2.37 (t, *J* = 8.1 Hz, 2H), 2.08 (s, 3H), 2.03 (m, 2H), 1.70 (t, *J* = 3.1 Hz, 3H), 1.28 (t, *J* = 7.1 Hz, 3H). $^{13}C\{^{1}H\}$ NMR (75 MHz, $CDCl_3$, DEPT) δ (ppm) 206.3 (C), 172.9 (C), 170.3 (C), 97.8 (C), 76.5 (CH_2), 73.4 (CH), 60.5 (CH_2), 30.3 (CH_2), 27.7 (CH_2), 21.0 (CH_3), 14.7 (CH_3), 14.2 (CH_3). HRMS (ESI/Q-TOF) *m/z*: $[M+H]^+$ calcd for $C_{12}H_{19}O_4$ 227.1283; found 227.1260.

Synthesis of (*R*)-2,6-dimethylocta-6,7-diene-2,5-diol ((+)-(*R*)-7)

A solution of ethyl (*R*)-4-acetoxy-5-methylhepta-5,6-dienoate ((+)-(*R*)-**9**, 60 mg, 0.27 mmol) in anhydrous Et_2O (1 mL) was slowly added to a solution of methylmagnesium bromide (3 M in Et_2O, 0.45 mL, 1.35 mmol) in anhydrous Et_2O (1.5 mL). The mixture was stirred under N_2 at room temperature for 5 h. The reaction was quenched with saturated NH_4Cl and extracted with ethyl acetate. The combined organic layer was washed with saturated $NaHCO_3$ and brine, dried with anhydrous $MgSO_4$. The solvent was evaporated in vacuum to give (+)-(*R*)-**7** (31.2 mg, 67%) $[\alpha]^{25}_{D}$ +17.5 (*c* 0.026, $CHCl_3$) as colorless oil.

Synthesis of the Mosher's derivatives of compound (−)-(*S*)-3

a) DCC (60 mg, 0.29 mmol), DMAP (7 mg, 0.06 mmol) and (*S*)-(−)-α-methoxy-α-(trifluoromethyl)phenylacetic acid (65 mg, 0.27 mmol) were added to a solution of (−)-(*S*)-**3** (21 mg, 0.11 mmol) in CH_2Cl_2 (7 mL) at 0 °C. The mixture was stirred at room temperature overnight. CH_2Cl_2 (15 mL) was added and the organic layer was washed with NaOH (2 N), HCl (5%) and brine. After drying over anhydrous $MgSO_4$, the solvent was removed in vacuum. The residue was purified by flash chromatography (*n*-hexane/EtOAc 8:2), to provide ethyl (*S*)-5-methyl-4-(((*S*)-3,3,3-trifluoro-2-methoxy-2-phenylpropanoyl)oxy)hepta-5,6-dienoate ((4*S,*2´*S*)-**8**) as a white solid (29 mg, 66%) and 5-(buta-2,3-dien-2-yl)dihydrofuran-2(3*H*)-one (**5**, 5 mg, 28%). Compound (4*S,2´S*)-**8**: $[\alpha]^{25}_D$ -138 (*c* 0.013, $CHCl_3$), ^1H NMR (600 MHz, $CDCl_3$) δ (ppm) 7.54 (m, 2H), 7.40 (m, 3H), 5.44 (t, *J* = 6.7 Hz, 1H), 4.82 (dqd, *J* = 10.8, 3.0, 1.2 Hz, 1H), 4.76 (dqd, *J* = 10.8, 3.0, 1.2 Hz, 1H), 4.12 (q, *J* = 7.1 Hz, 2H), 3.55 (s, 3H), 2.24 (m, 2H), 2.03 (m, 2H), 1.70 (t, *J* = 3.2 Hz, 3H), 1.25 (t, *J* = 7.1 Hz, 3H). $^{13}C\{^1H\}$ NMR (75 MHz, $CDCl_3$, DEPT) δ (ppm) 207.0 (C), 172.5 (C), 165.9 (C), 132.3 (C), 129.6 (CH), 128.4 (CH), 127.4 (CH), 125.3 (C, q, $J_{C\text{-}F}$ = 287 Hz), 96.9 (C), 84.6 (C, q, $J_{C\text{-}F}$ = 27 Hz), 77.0 (CH_2), 76.9 (CH), 60.6 (CH_2), 55.5 (CH_3), 29.8 (CH_2), 27.6 (CH_2), 14.6 (CH_3), 14.2 (CH_3). ^{19}F NMR (282 MHz, $CDCl_3$) δ (ppm) -71.31 (s).

b) The same protocol was repeated using (*R*)-(−)-α-methoxy-α-(trifluoromethyl)phenylacetic acid. Ethyl (*S*)-5-methyl-4-(((*R*)-3,3,3-trifluoro-2-methoxy-2-phenylpropanoyl)oxy)hepta-5,6-dienoate ((4*S,*2´*R*)-**8**, 32 mg, 73%) and 5-(buta-2,3-dien-2-yl)dihydrofuran-2(3*H*)-one (**5**, 4 mg, 22%) were obtained. Compound (4*S,2´R*)-**8**: $[\alpha]^{25}_D$ +10.1 (*c* 0.0063, $CHCl_3$), ^1H NMR (600 MHz, $CDCl_3$) δ (ppm) 7.53 (m, 2H), 7.40 (m, 3H), 5.42 (t, *J* = 6.8 Hz, 1H), 4.79 (m, 1H), 4.67 (m, 1H), 4.13 (q, *J* = 7.2 Hz, 2H), 3.55 (s, 3H), 2.34 (m, 2H), 2.09 (q, *J* = 7.8 Hz, 2H), 1.58 (t, *J* = 3.6 Hz, 3H), 1.25 (t, *J* = 7.2 Hz, 3H).). $^{13}C\{^1H\}$ NMR (75 MHz, $CDCl_3$, DEPT) δ (ppm) 207.0 (C), 172.5 (C), 165.9 (C), 132.2 (C), 129.6 (CH), 128.4 (CH), 127.4 (CH), 127.0 (C, q, $J_{C\text{-}F}$ = 287 Hz), 96.7

(C), 84.6 (C, q, J_{C-F} = 27 Hz), 77.2 (CH_2), 76.8 (CH), 60.7 (CH_2), 55.5 (CH_3), 30.1 (CH_2), 27.6 (CH_2), 14.2 (CH_3). ^{19}F NMR (282 MHz, $CDCl_3$) δ (ppm) -71.50 (s).

Synthesis of the Mosher's derivative of racemic compound (±)-3

Compound **3** (15.8 mg, 0.09 mmol), DCC (46 mg, 0.22 mmol), DMAP (5.50 mg, 0.045 mmol) and (*S*)-(−)-α-methoxy-α-(trifluoromethyl)phenylacetic acid (53.70 mg, 0.26 mmol), according to mentioned procedure, afforded compound **8** (11 mg, 31%) (as a mixture of inseparable isomers (4*S*,2´*S*)-8, and (4*R*,2´*S*)-8)), and 5-(buta-2,3-dien-2-yl)dihydrofuran-2(3*H*)-one (**5**, 5.4 mg, 43%).

References

[1] Smith, A. B.; Fukui, M.; Vaccaro, H. A.; Empfield, J. R. *J. Am. Chem. Soc* **1991**, *113* (6), 2071-2092. https://doi.org/10.1021/ja00006a029
[2] Li, W.; Lin, Z.; Chen, L.; Tian, X.; Wang, Y.; Huang, S.-H.; Hong, R. *Tetrahedron Lett.* **2016**, *57* (5), 603-606. https://doi.org/10.1016/j.tetlet.2015.12.098

NMR and IR spectra

^{1}H NMR, DEPT 135, ^{13}C NMR, HSQC, HMBC and IR of 3-methyl-2-(3-methylbut-2-en-1-yl)-2,5-dihydrofuran (**1**)

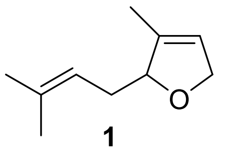

1

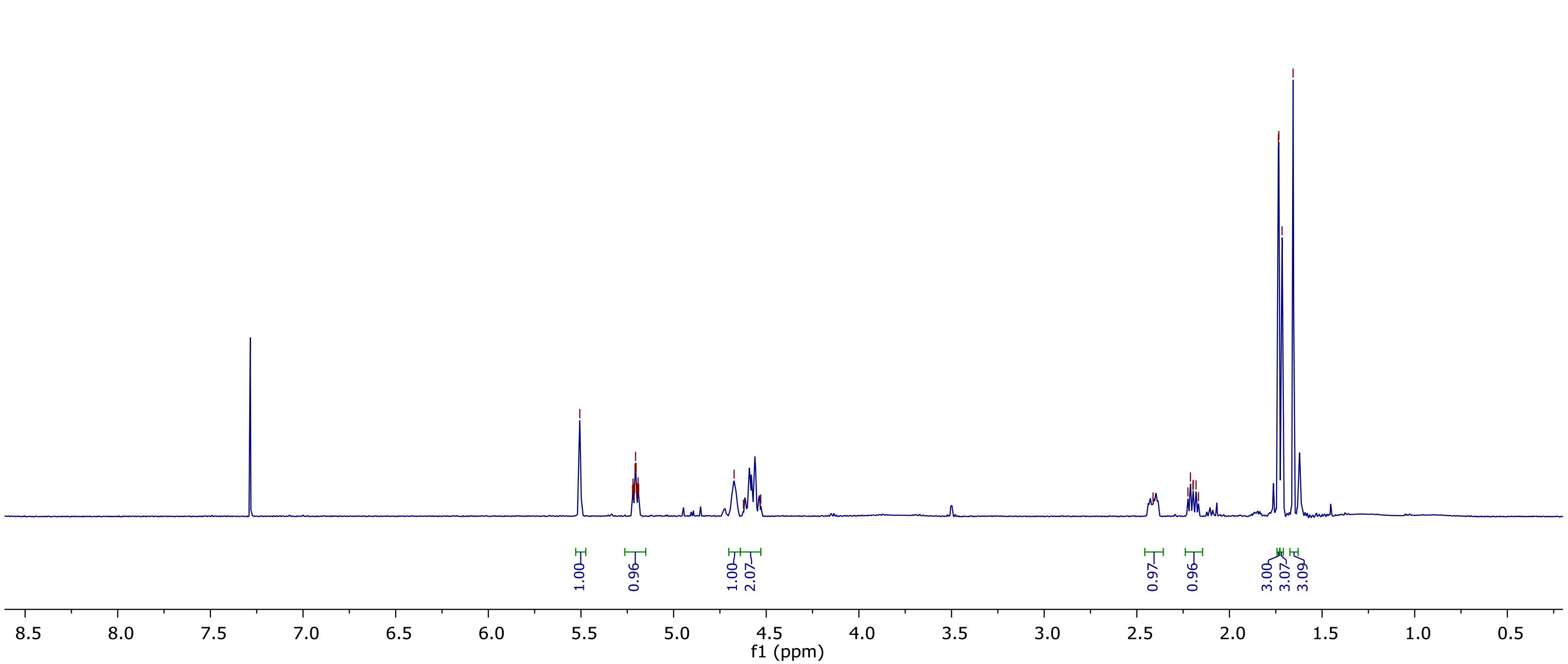

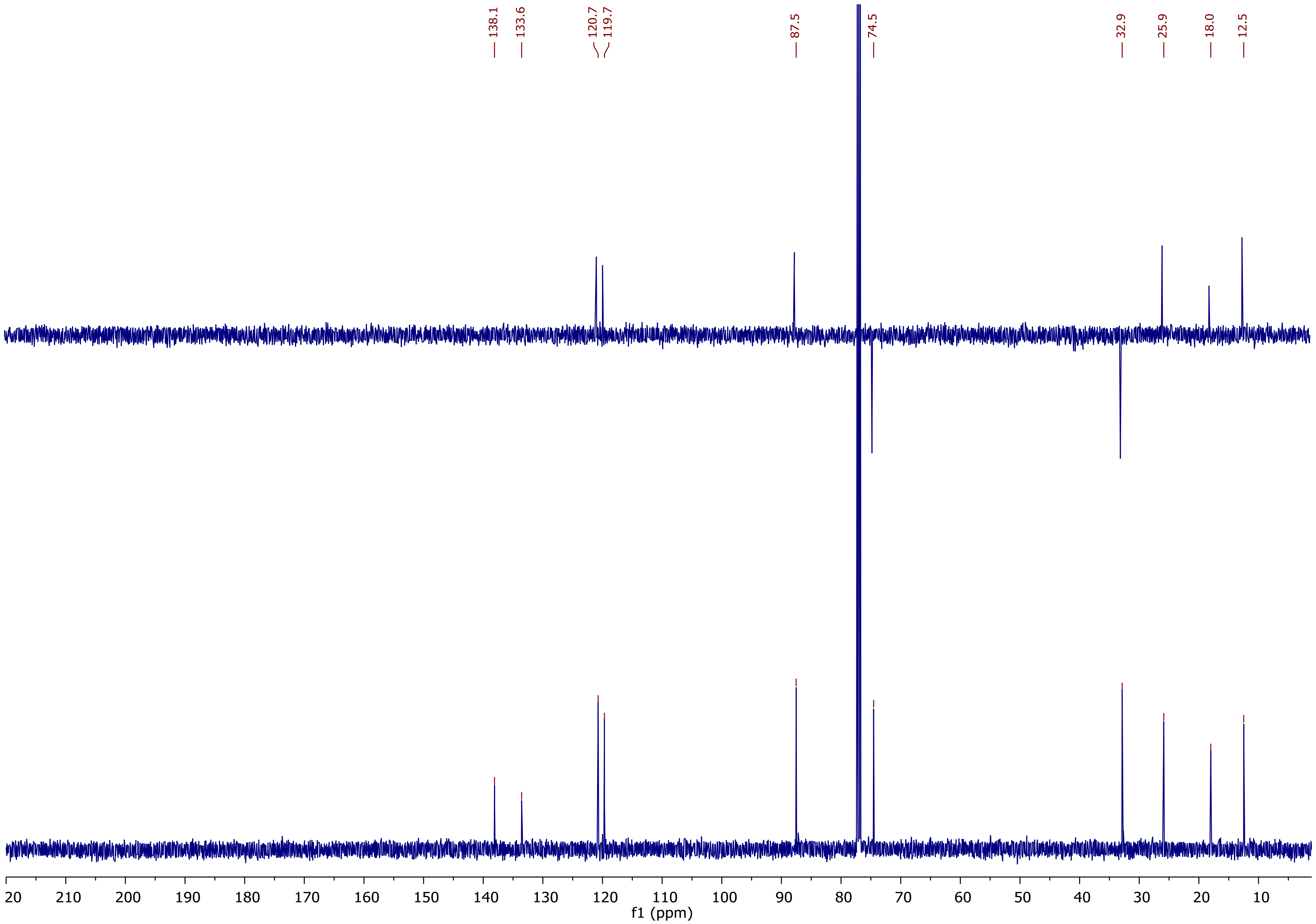
138.1
133.6
120.7
119.7
87.5
74.5
32.9
25.9
18.0
12.5
f1 (ppm)

HSQC

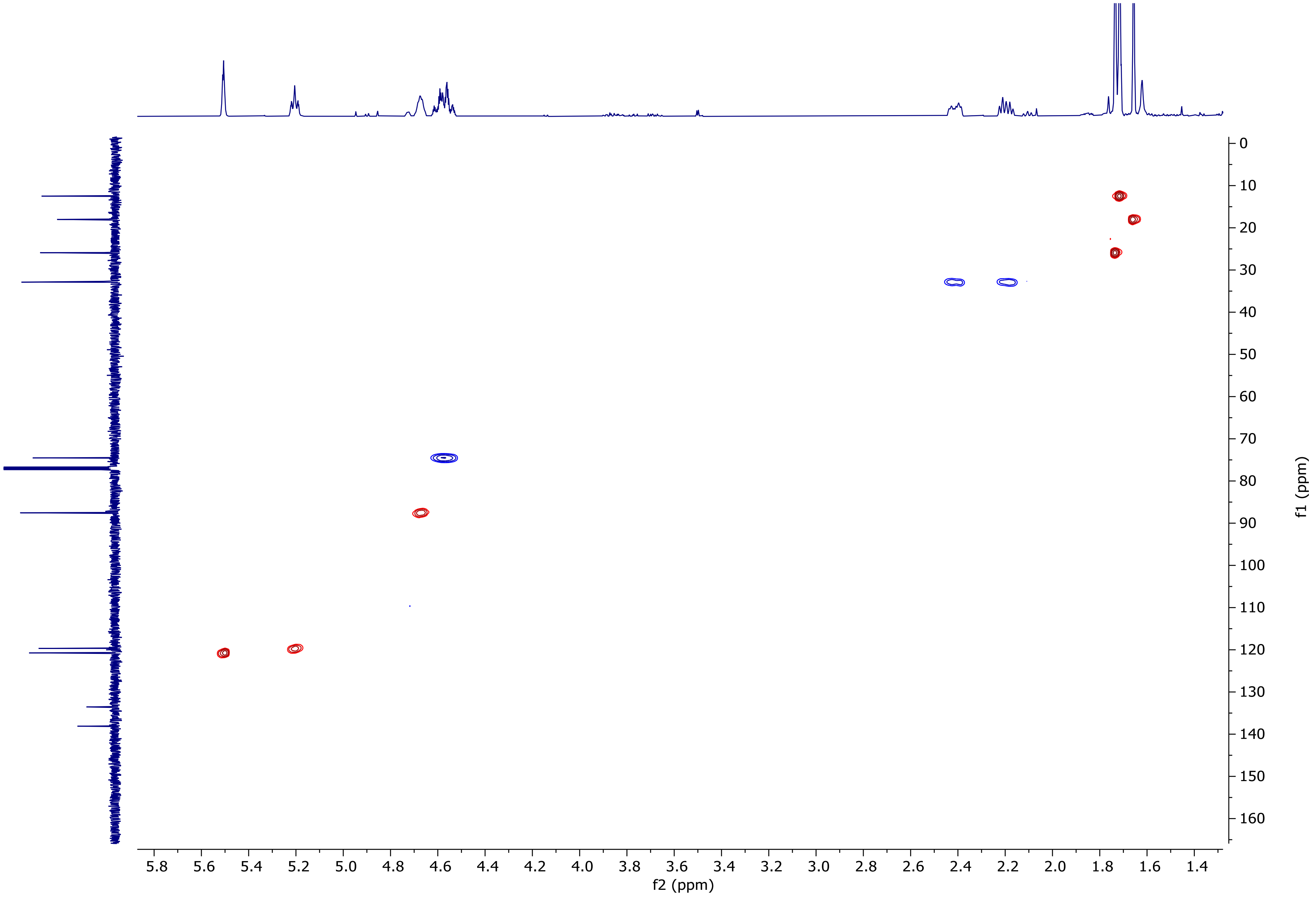

5.8 5.6 5.4 5.2 5.0 4.8 4.6 4.4 4.2 4.0 3.8 3.6 3.4 3.2 3.0 2.8 2.6 2.4 2.2 2.0 1.8 1.6 1.4
f2 (ppm)
0 10 20 30 40 50 60 70 80 90 100 110 120 130 140 150 160
f1 (ppm)

HMBC

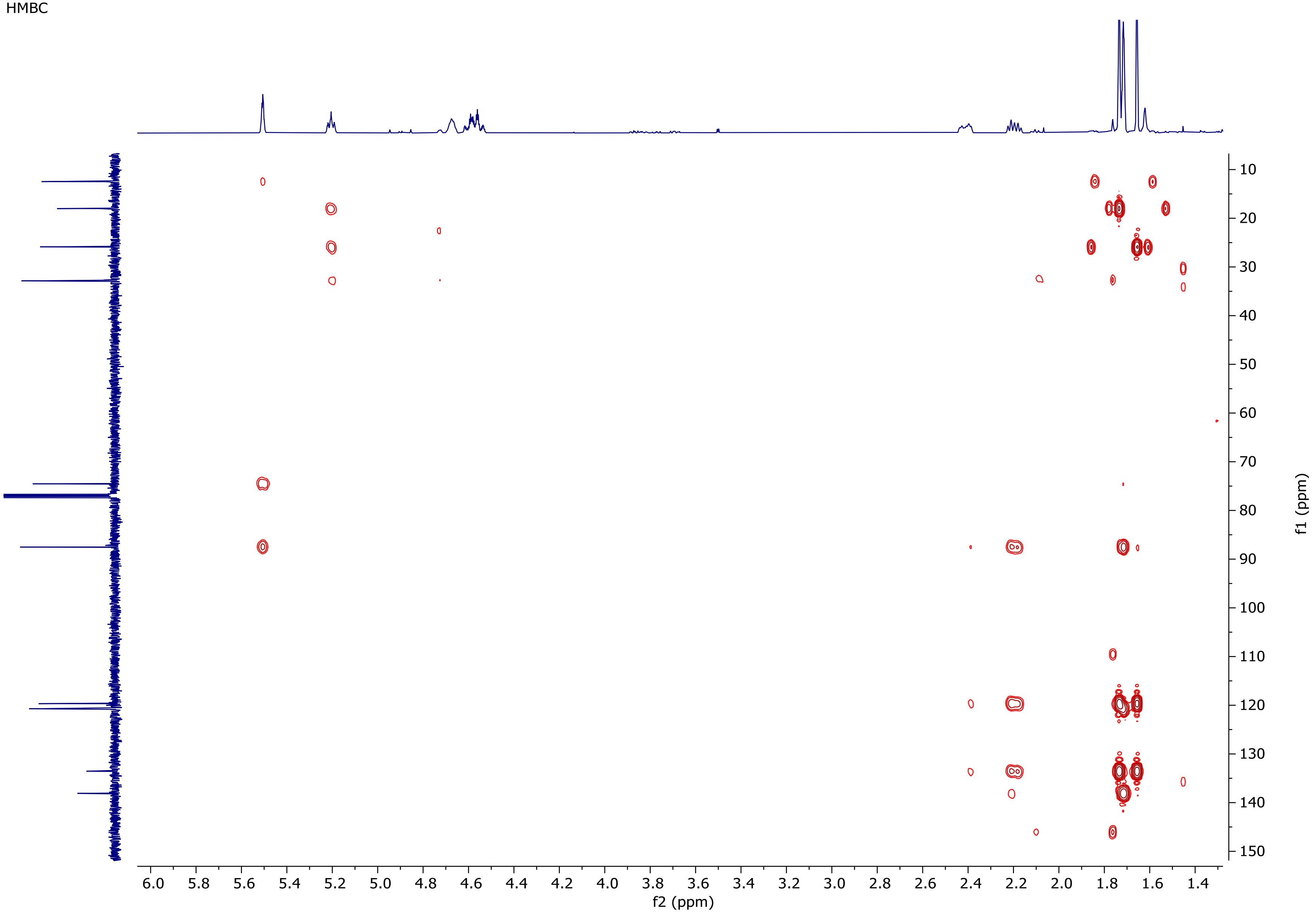

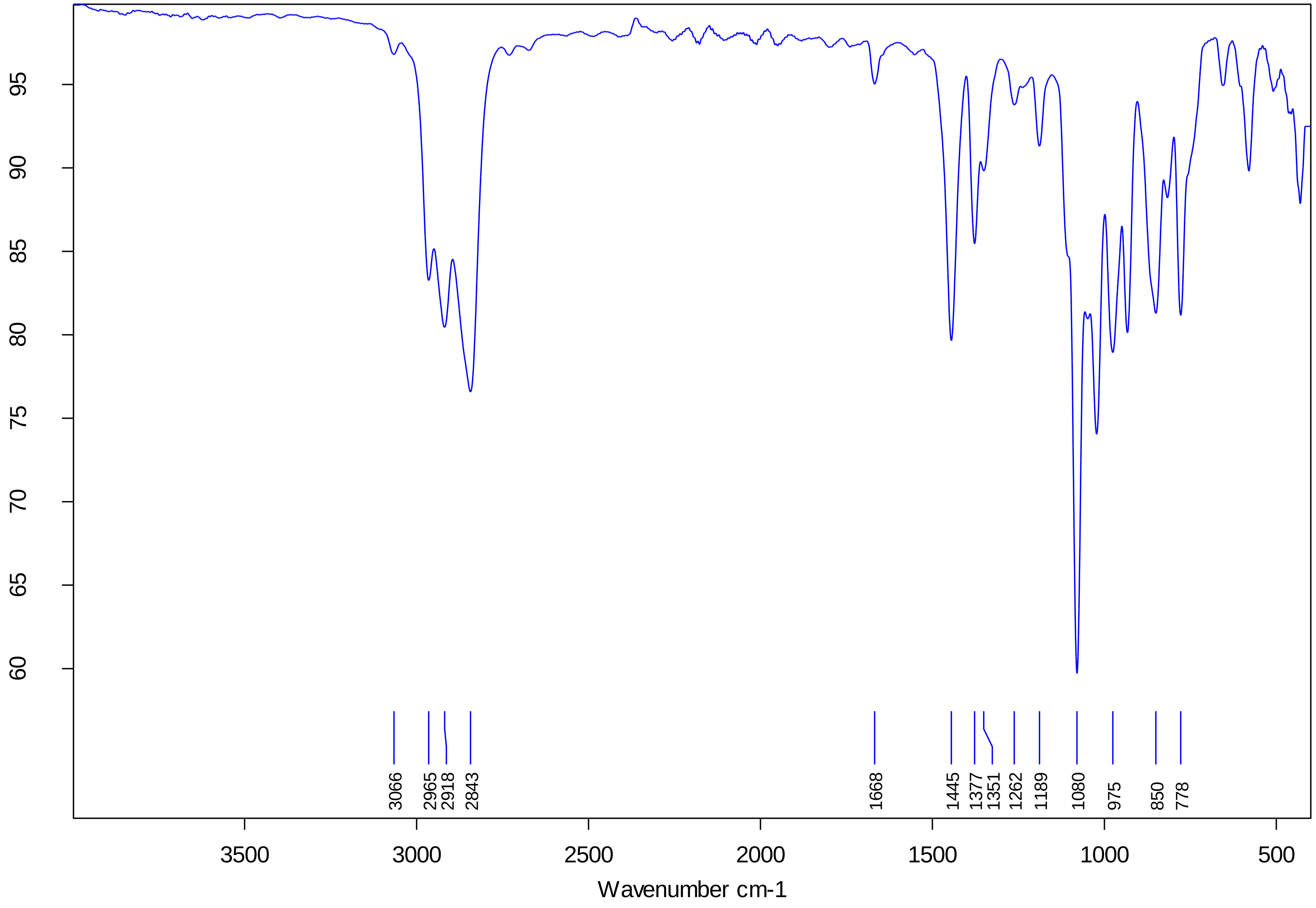

95
90
85
80
75
70
65
60
3066
2965
2918
2843
1668
1445
1377
1351
1262
1189
1080
975
850
778
3500
3000
2500
2000
1500
1000
500
Wavenumber cm-1

^{1}H NMR, DEPT 135, ^{13}C NMR and IR of ethyl 3-(3-methyl-2,5-dihydrofuran-2-yl)propanoate (**2**)

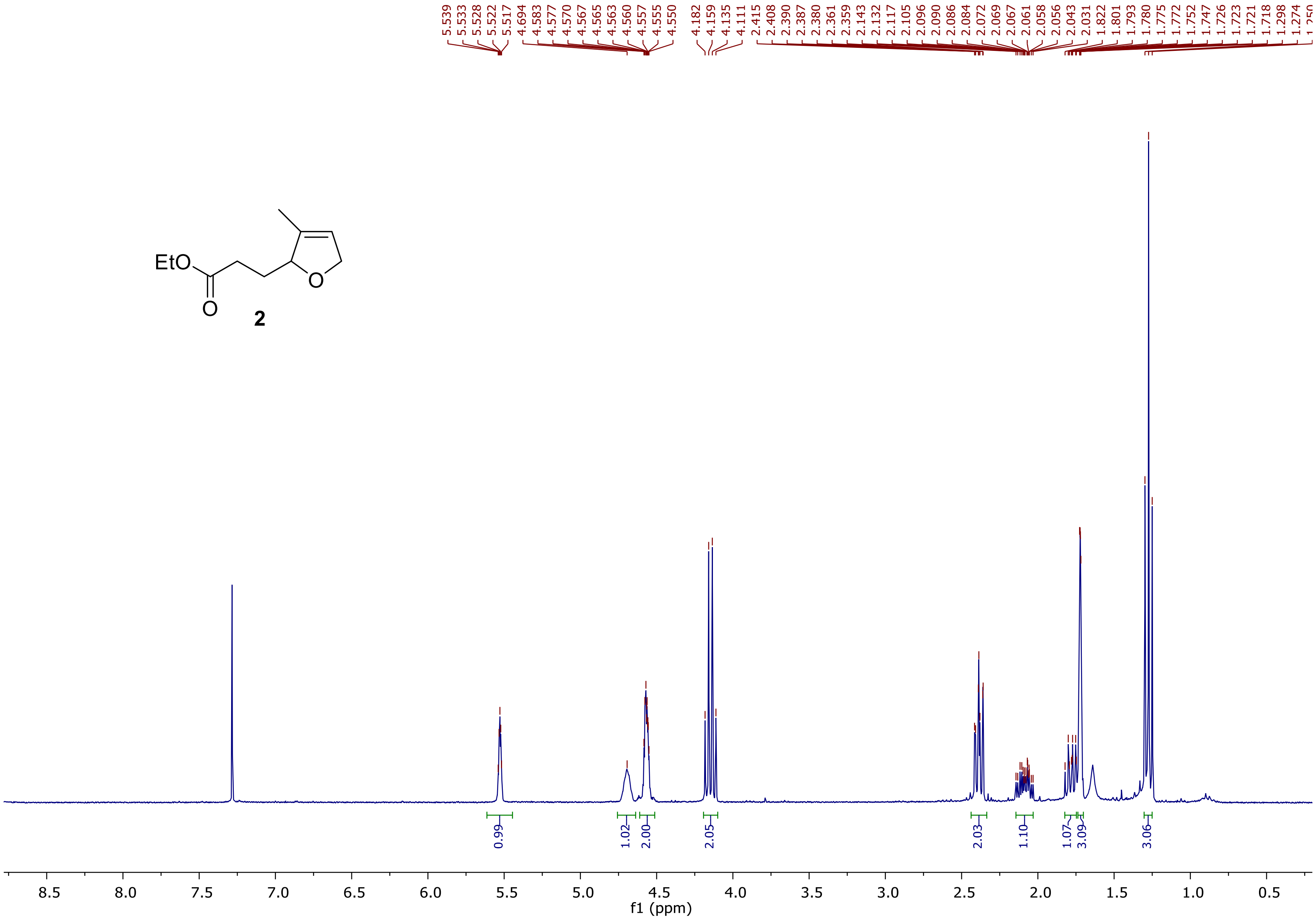

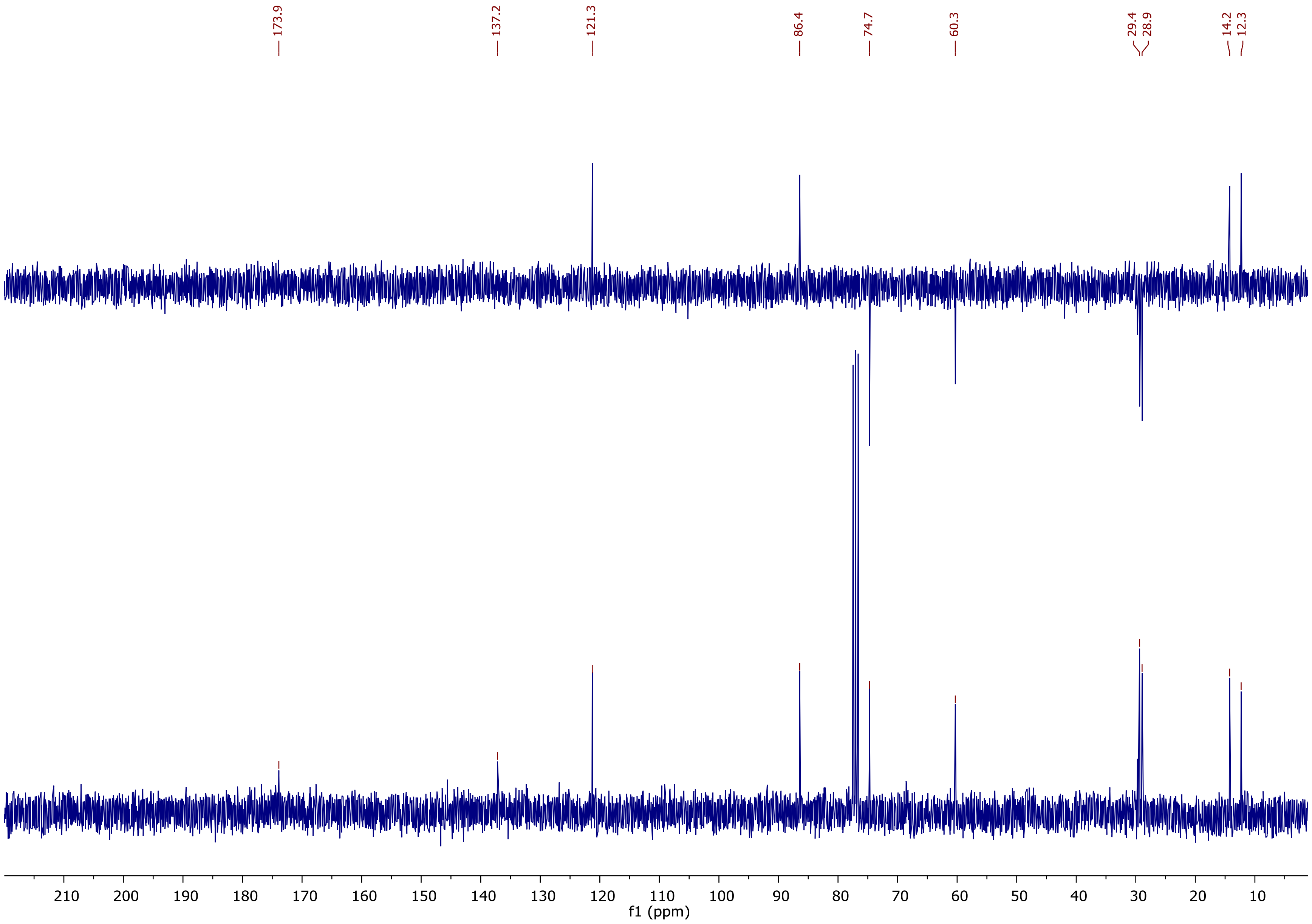

173.9
137.2
121.3
86.4
74.7
60.3
29.4
28.9
14.2
12.3
210 200 190 180 170 160 150 140 130 120 110 100 90 80 70 60 50 40 30 20 10
f1 (ppm)

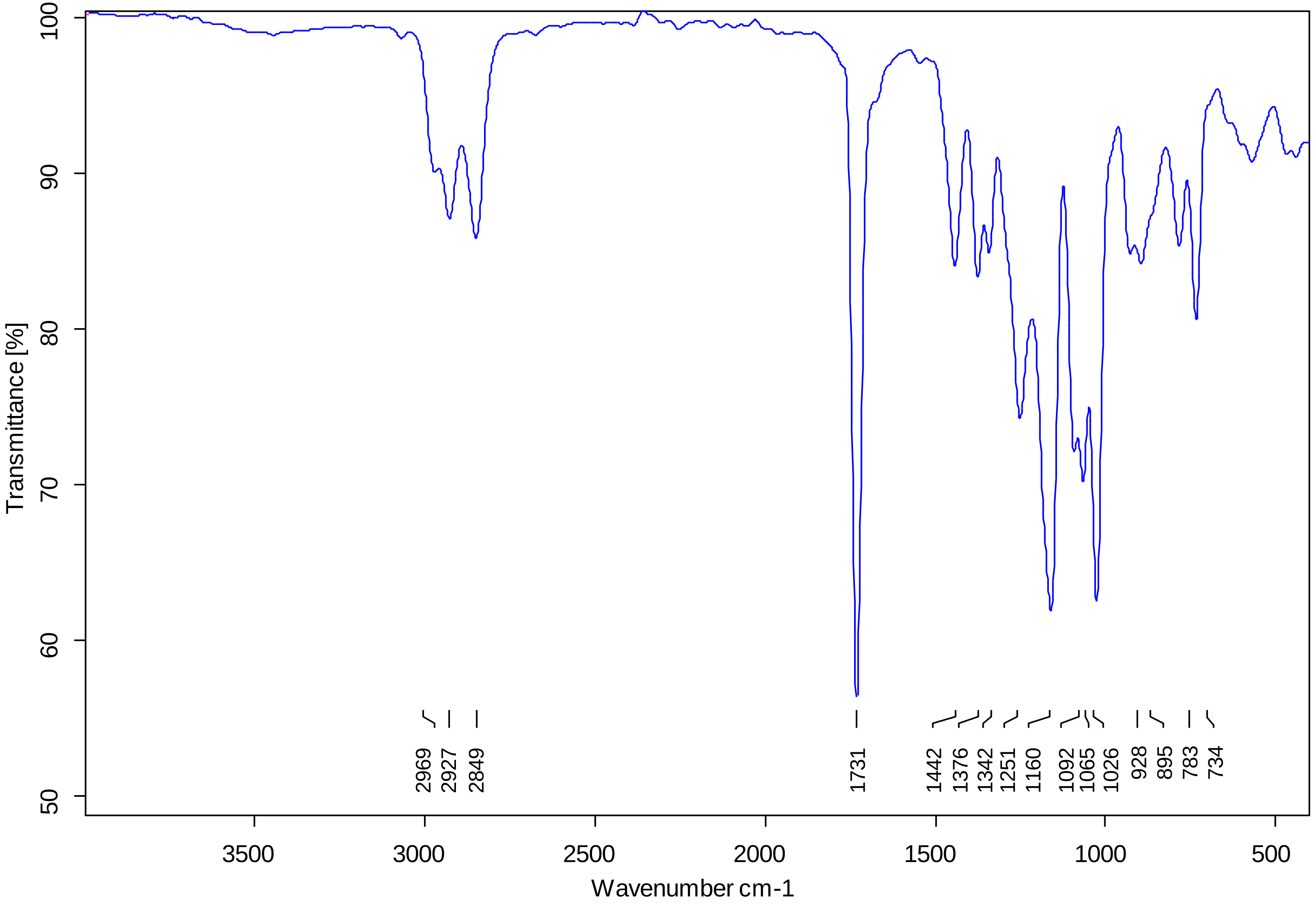

100
90
80
70
60
50
Transmittance [%]
2969
2927
2849
1731
1442
1376
1342
1251
1160
1092
1065
1026
928
895
783
734
3500
3000
2500
2000
1500
1000
500
Wavenumber cm-1

^{1}H NMR, DEPT 135, ^{13}C NMR and IR of ethyl 4-hydroxy-5-methylhepta-5,6-dienoate (**3**)

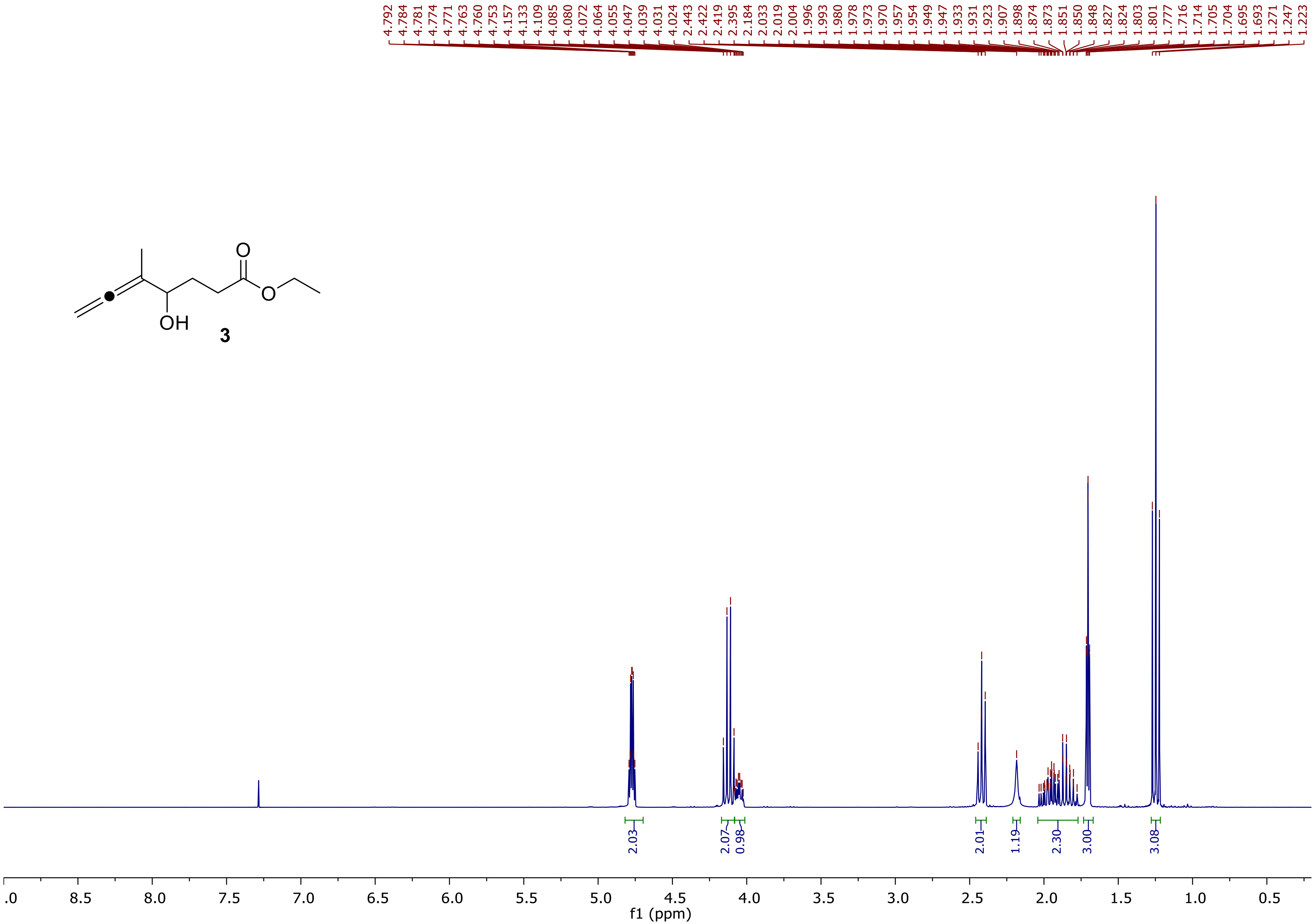

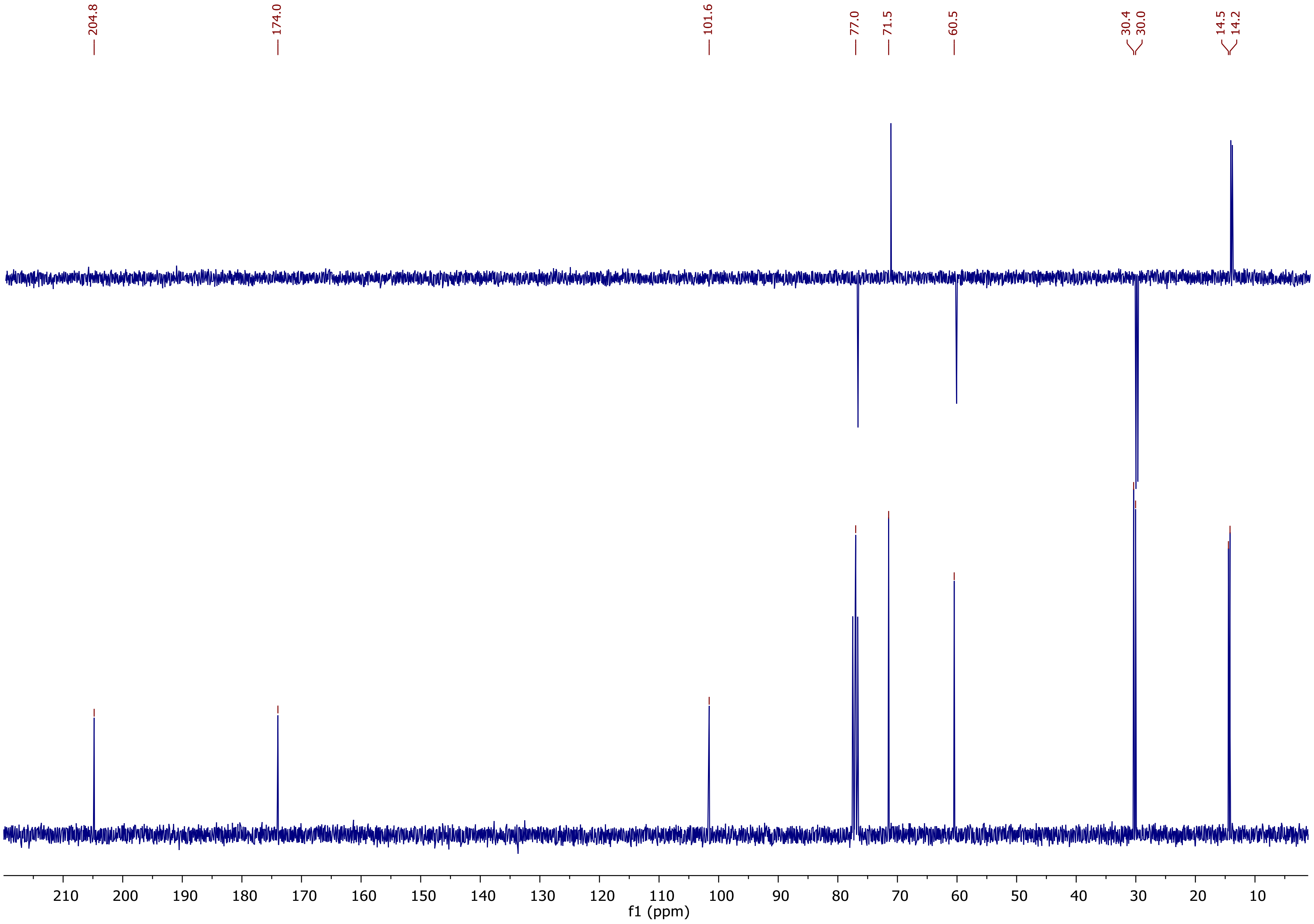
204.8
174.0
101.6
77.0
71.5
60.5
30.4
30.0
14.5
14.2
210 200 190 180 170 160 150 140 130 120 110 100 90 80 70 60 50 40 30 20 10
f1 (ppm)

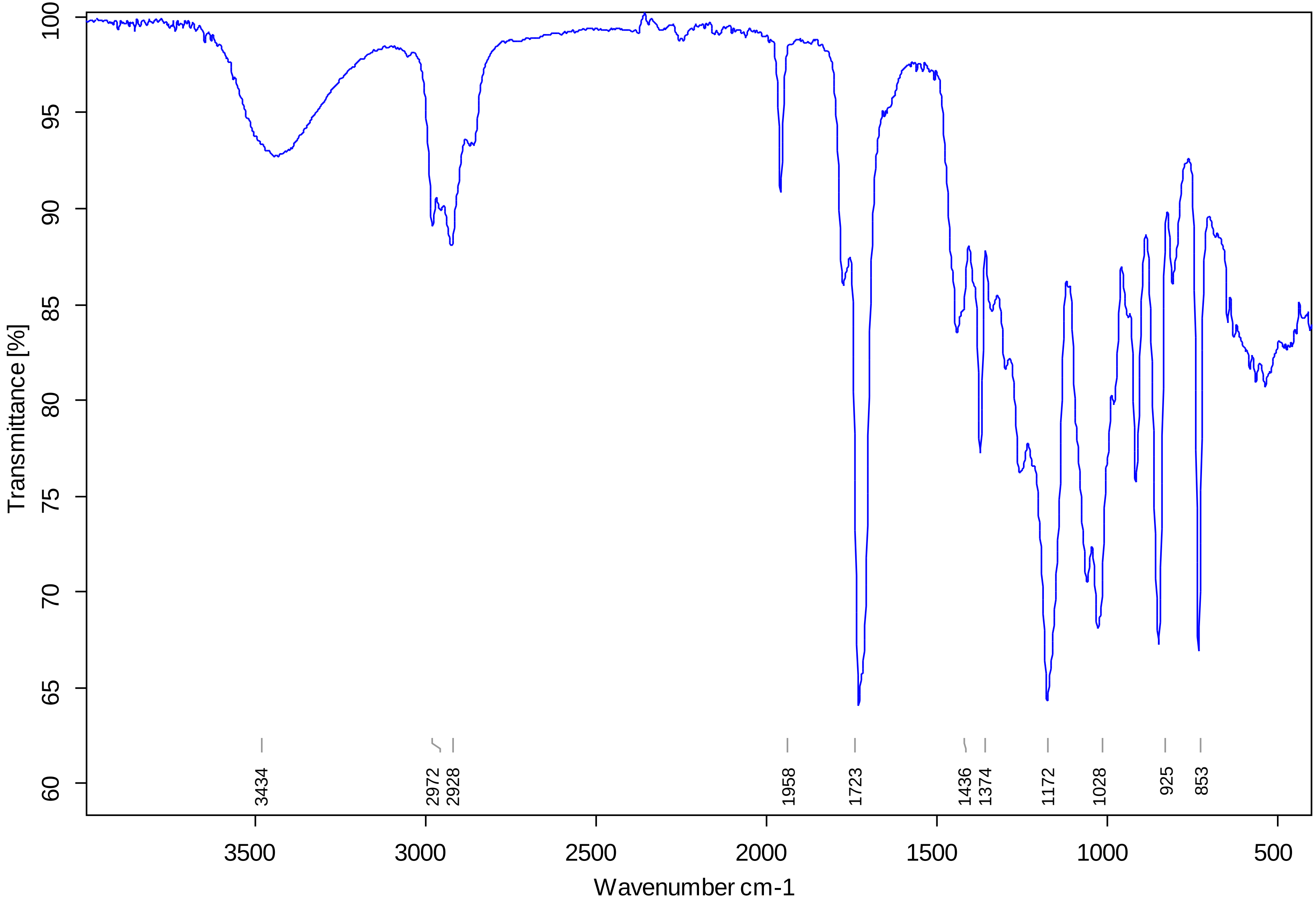
Transmittance [%]
Wavenumber cm-1
3434
2972
2928
1958
1723
1436
1374
1172
1028
925
853
100
95
90
85
80
75
70
65
60
3500
3000
2500
2000
1500
1000
500

^{1}H NMR, DEPT 135, ^{13}C NMR and IR of ethyl 4-oxobutanoate (**4**)

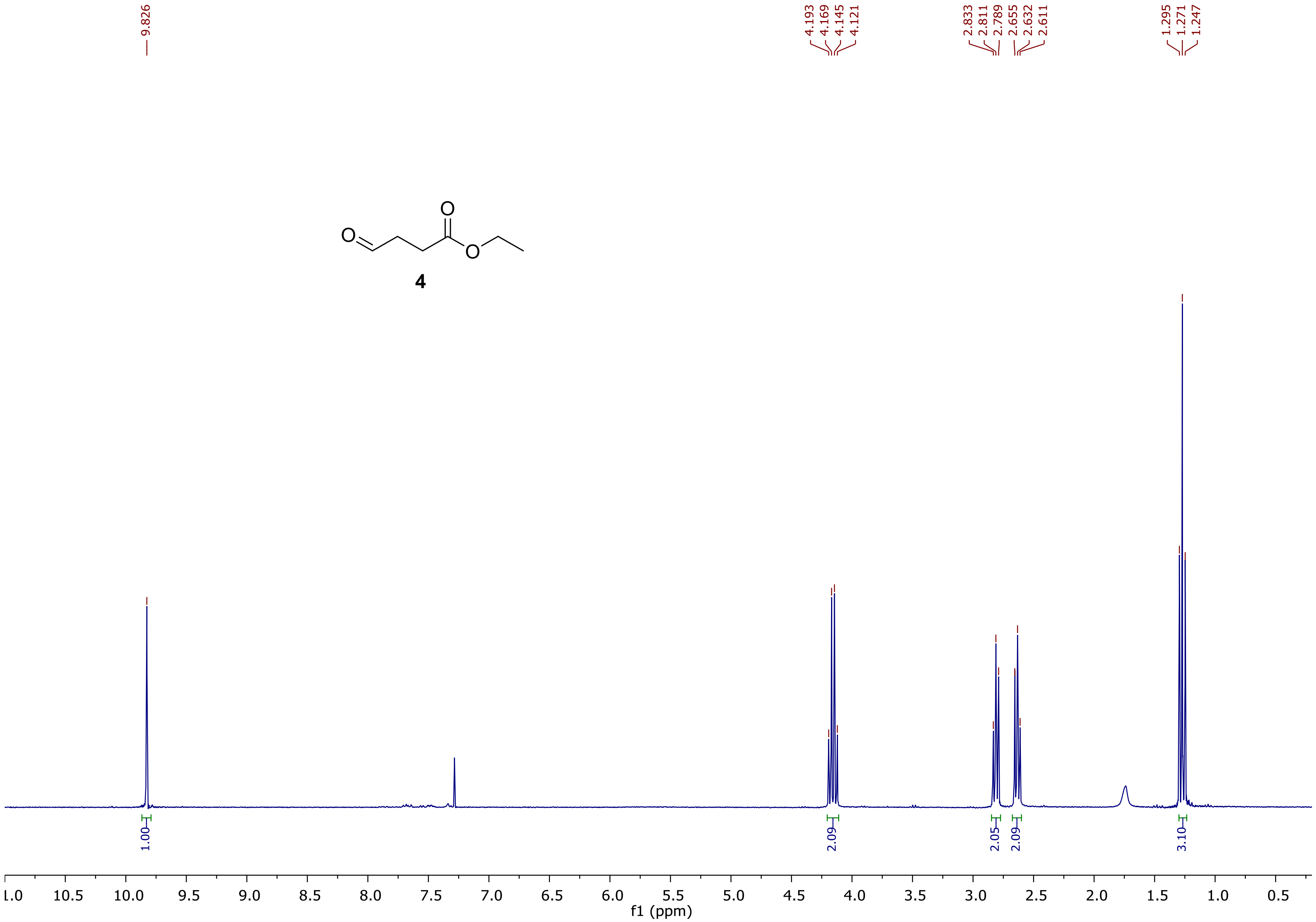

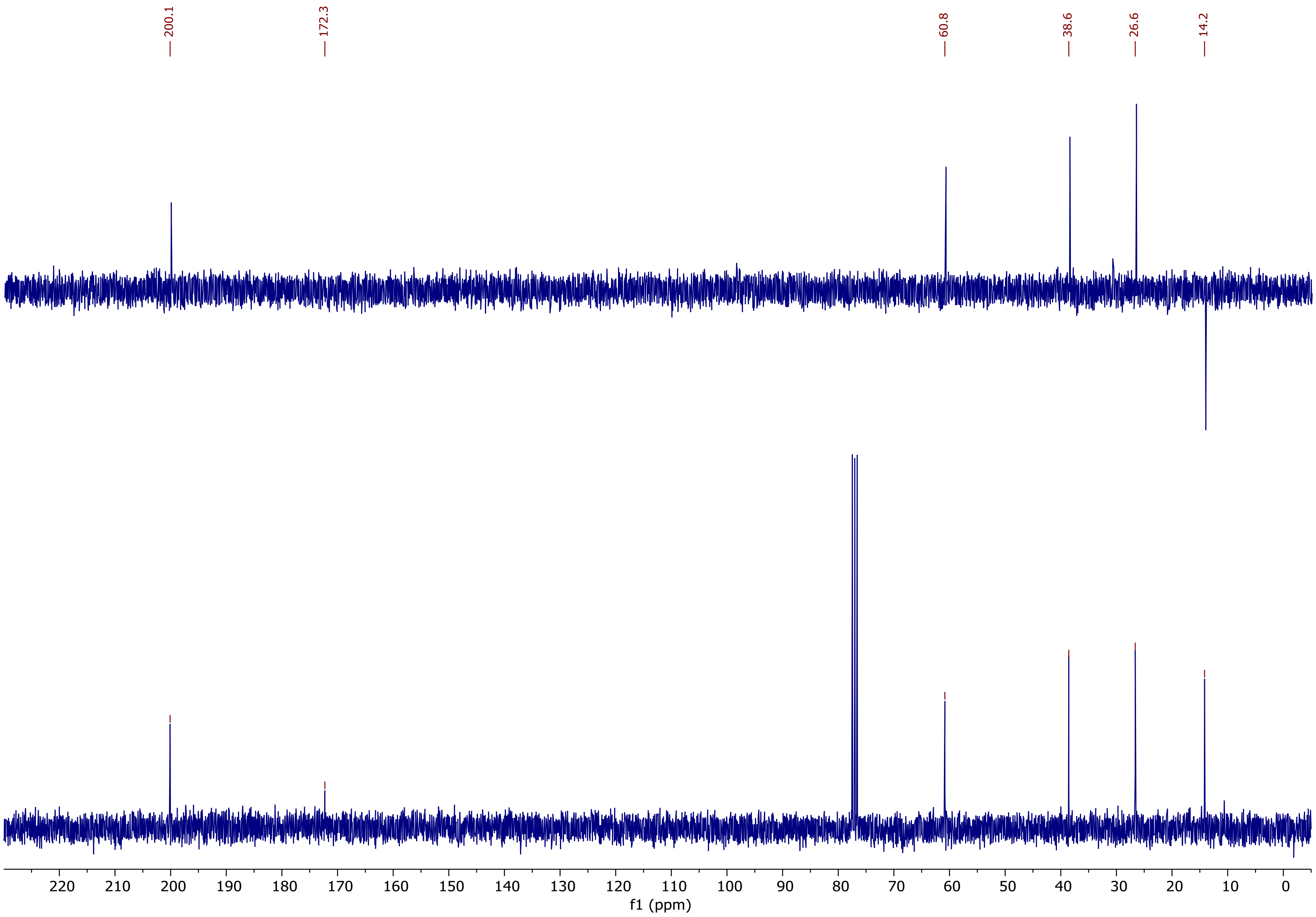
200.1
172.3
60.8
38.6
26.6
14.2
220
210
200
190
180
170
160
150
140
130
120
110
100
90
80
70
60
50
40
30
20
10
0
f1 (ppm)

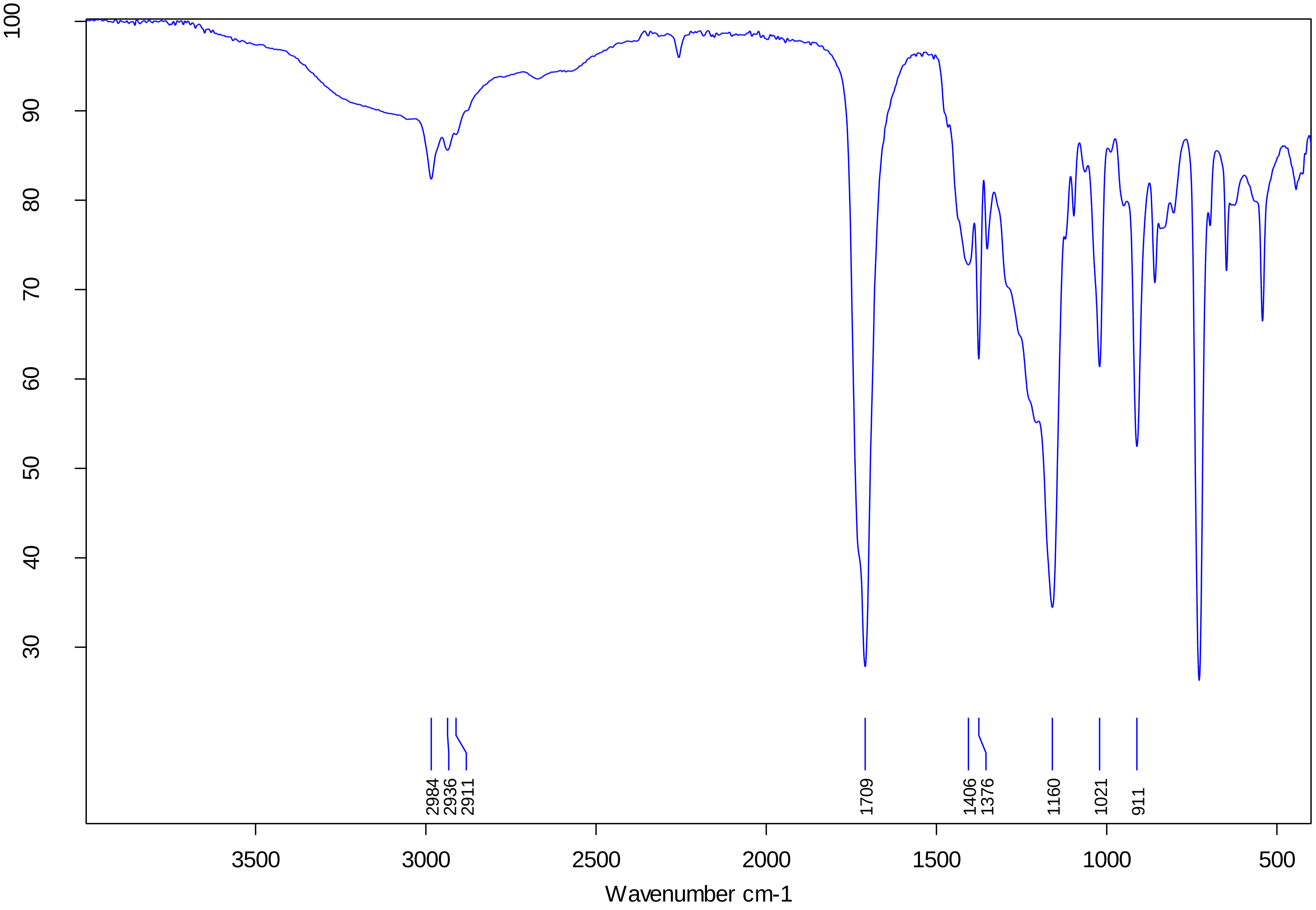
100
90
80
70
60
50
40
30
2984
2936
2911
1709
1406
1376
1160
1021
911
3500
3000
2500
2000
1500
1000
500
Wavenumber cm-1

^{1}H NMR, DEPT 135, ^{13}C NMR and IR of 5-(buta-2,3-dien-2-yl)dihydrofuran-2(3*H*)-one (**5**)

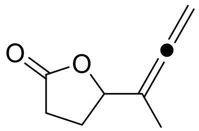

5

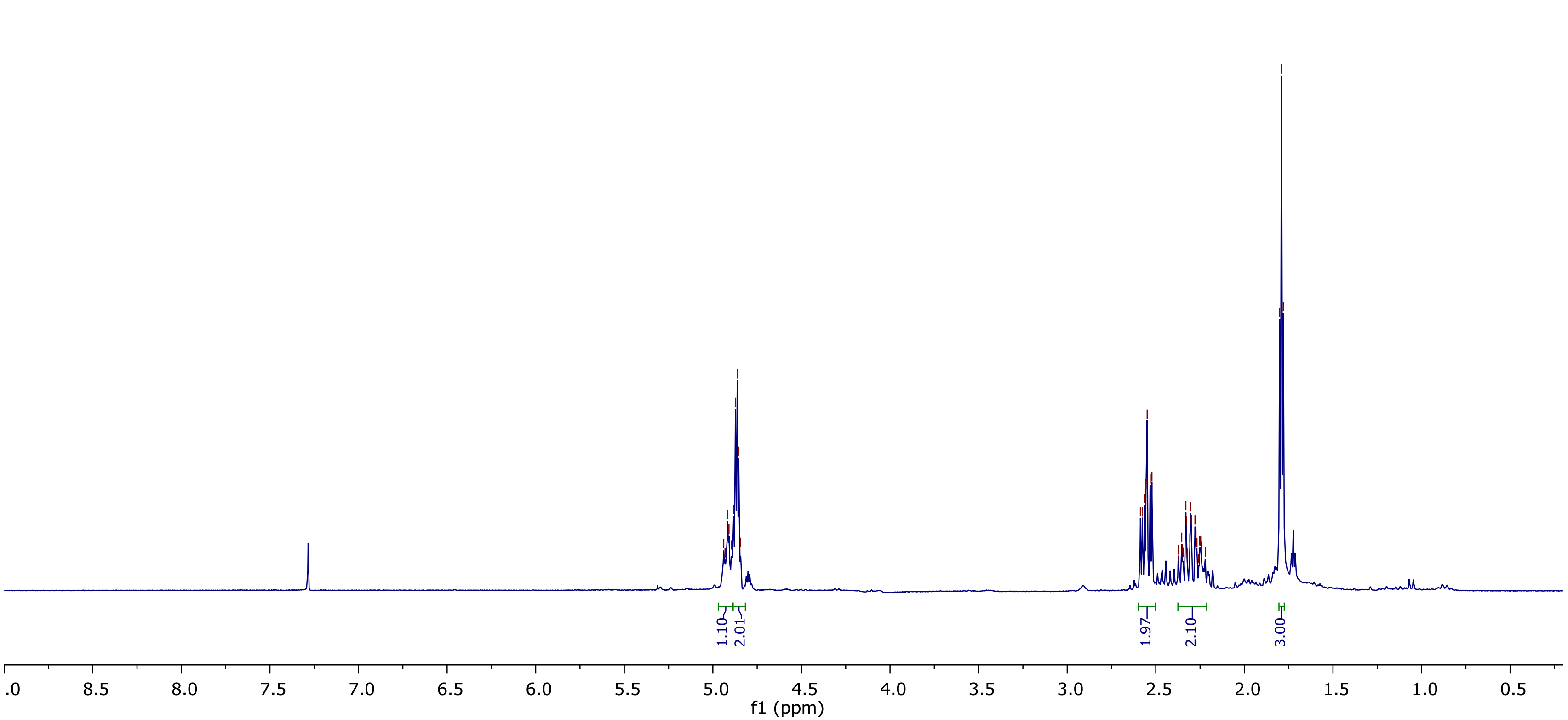

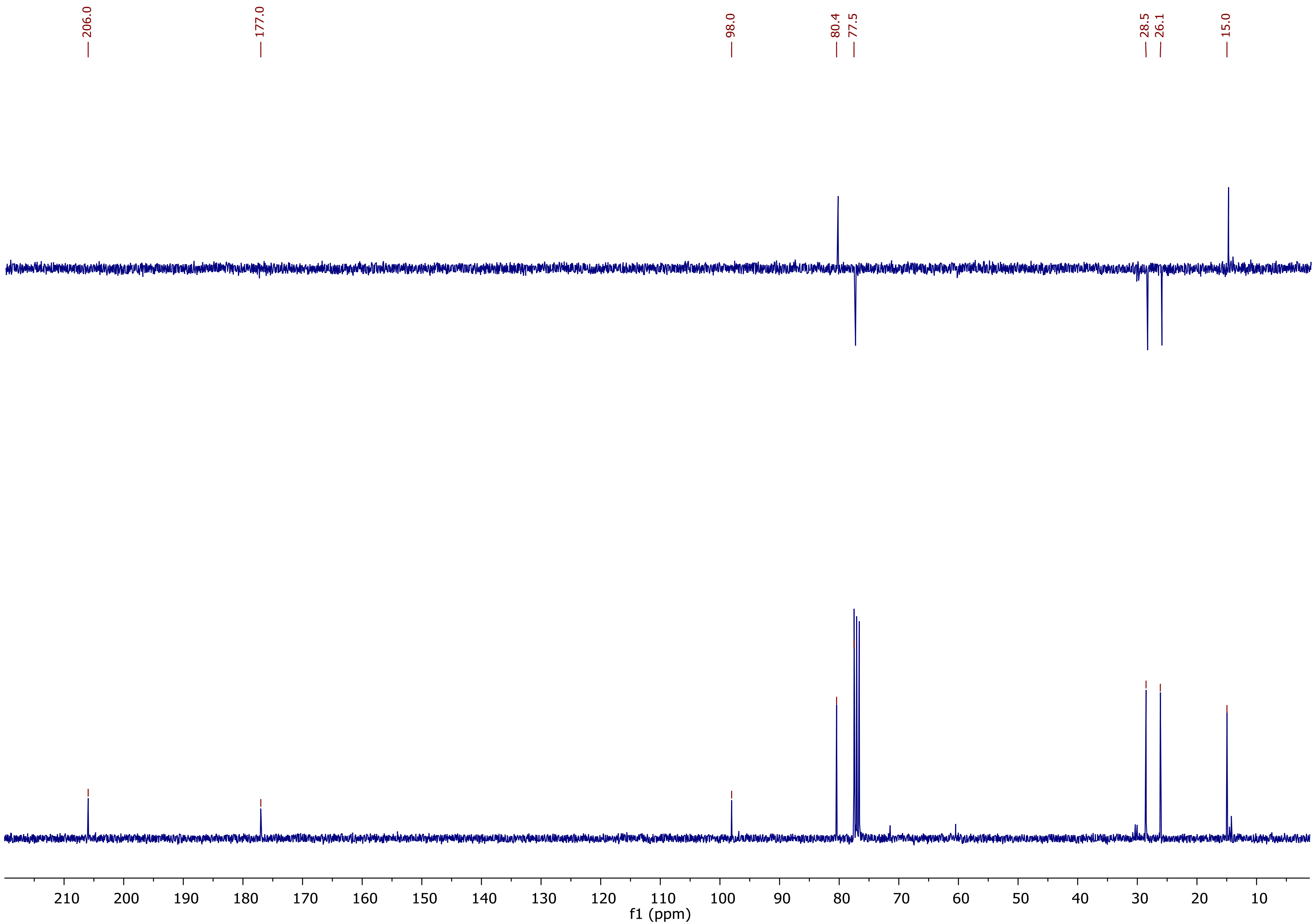
206.0
177.0
98.0
80.4
77.5
28.5
26.1
15.0
210 200 190 180 170 160 150 140 130 120 110 100 90 80 70 60 50 40 30 20 10
f1 (ppm)

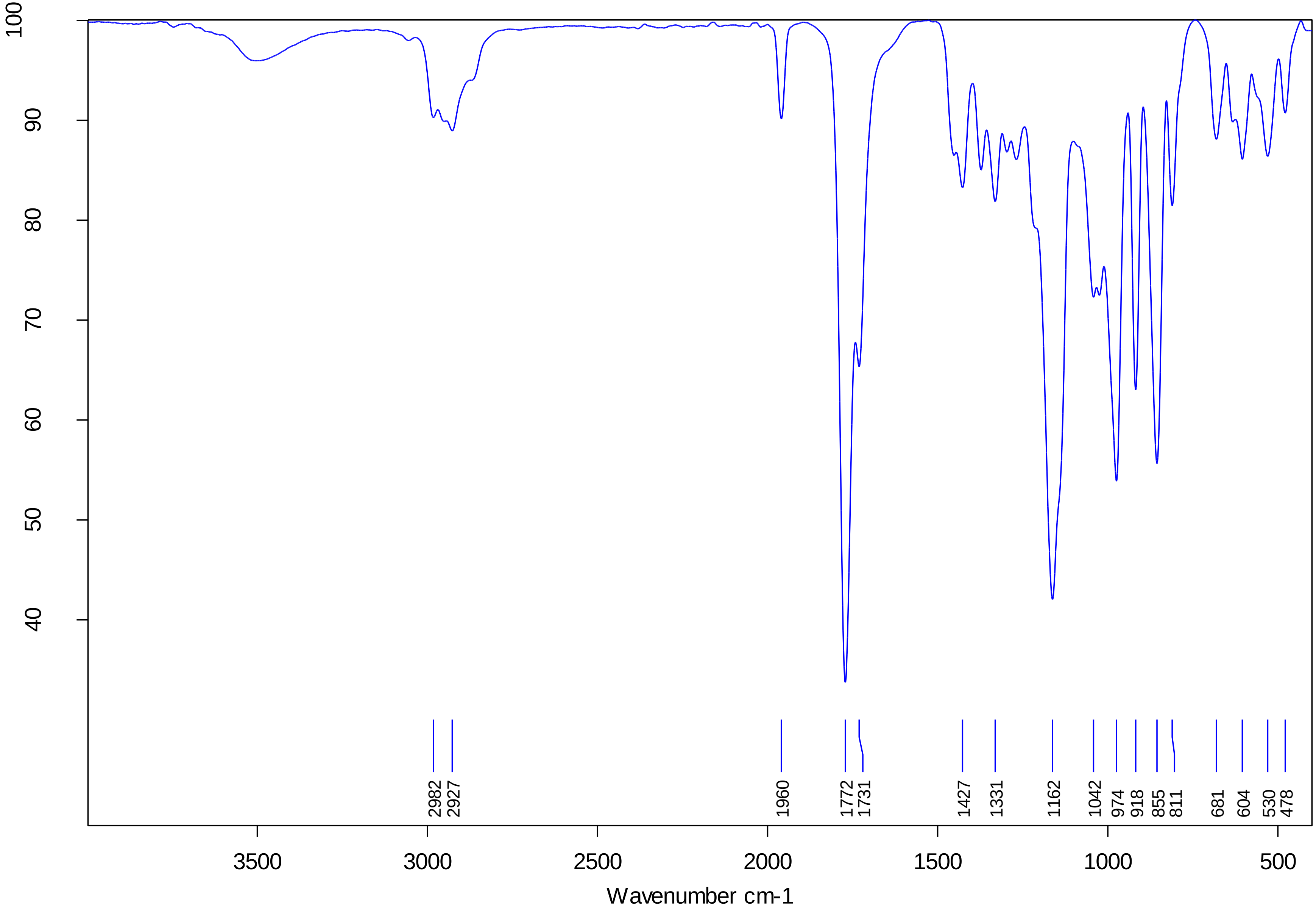
100
90
80
70
60
50
40
2982
2927
1960
1772
1731
1427
1331
1162
1042
974
918
855
811
681
604
530
478
3500
3000
2500
2000
1500
1000
500
Wavenumber cm-1

^{1}H NMR, DEPT 135, ^{13}C NMR and IR of 2-methyl-4-(3-methyl-2,5-dihydrofuran-2-yl)butan-2-ol (**6**)

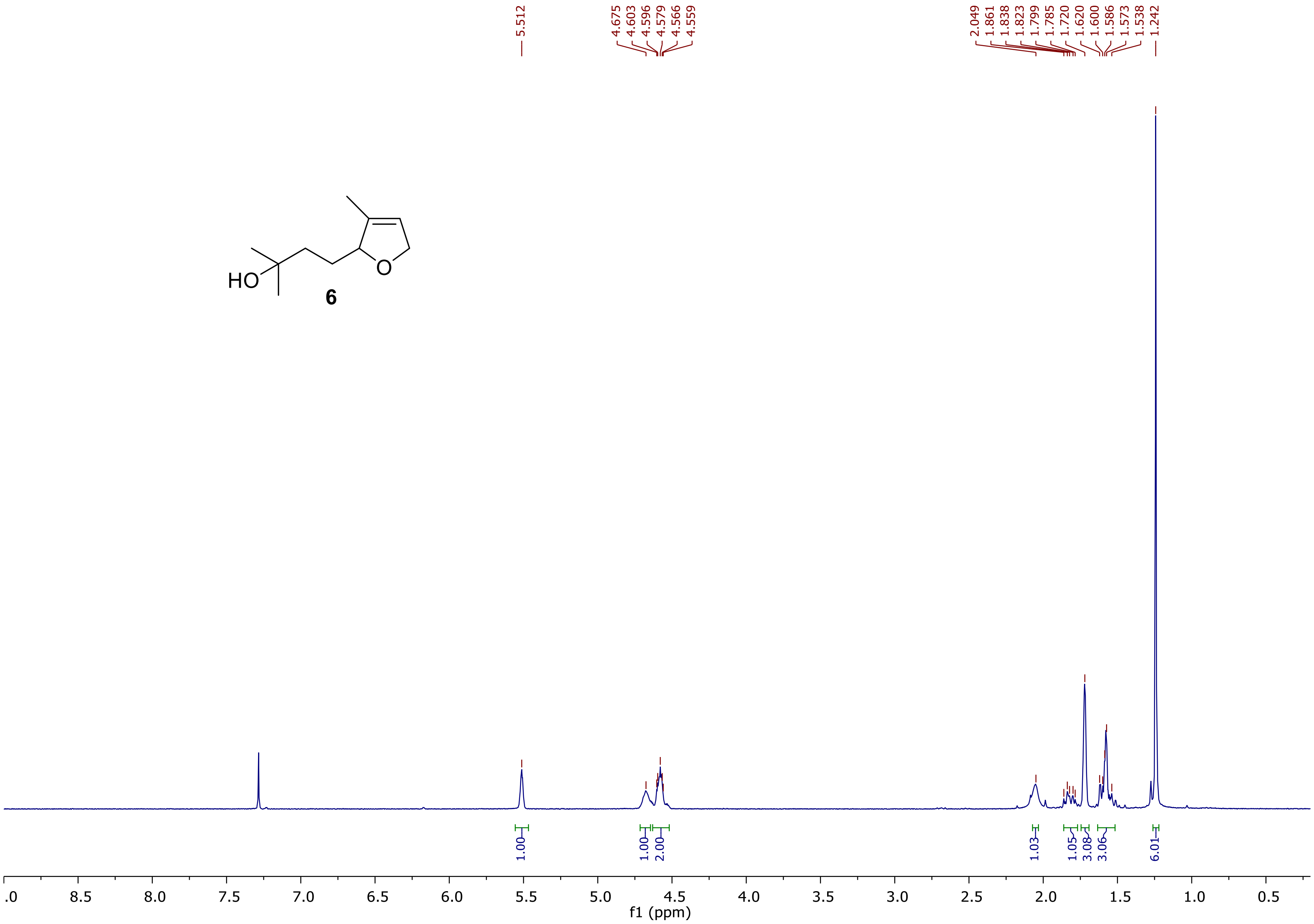

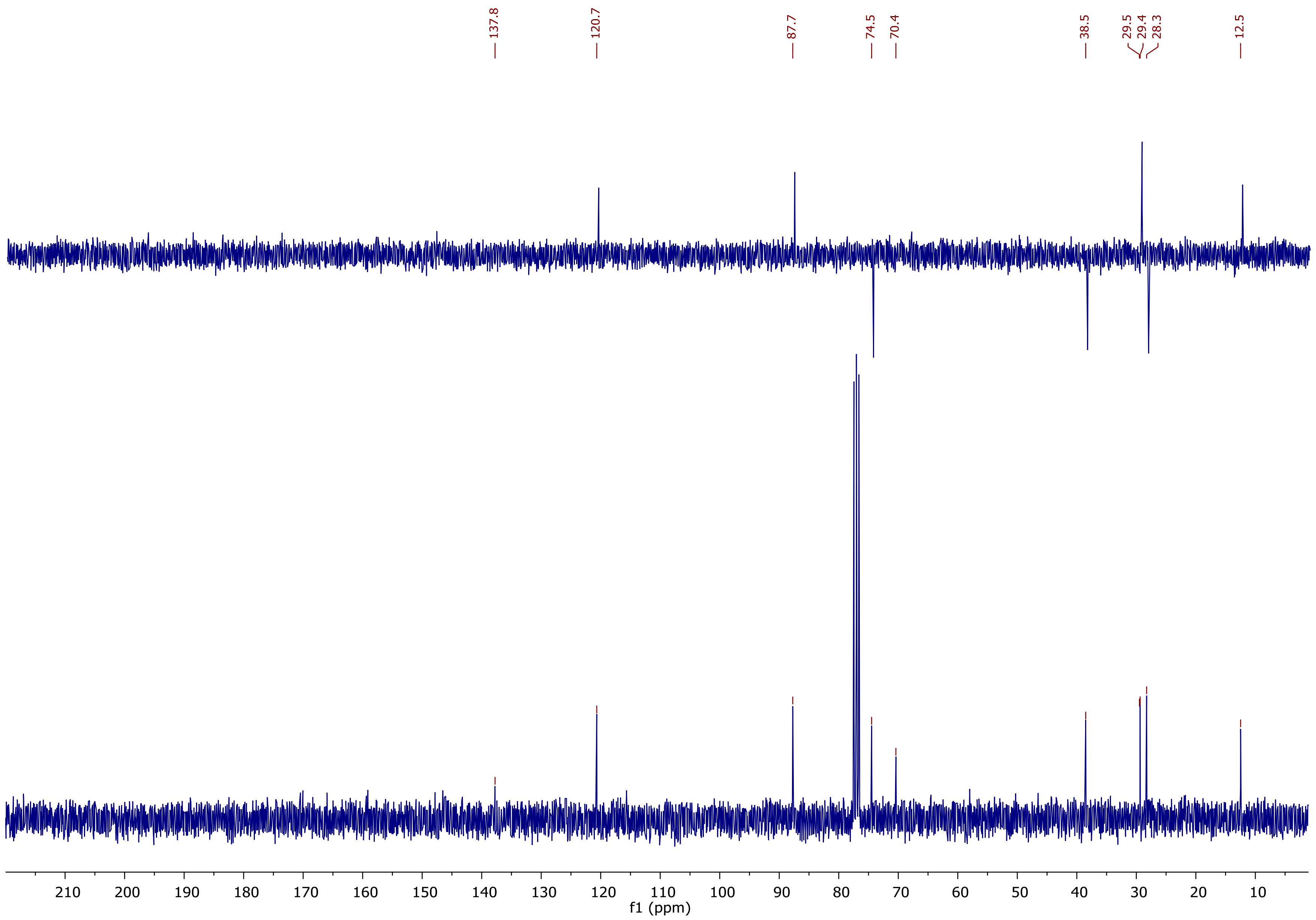
137.8
120.7
87.7
74.5
70.4
38.5
29.5
29.4
28.3
12.5
210
200
190
180
170
160
150
140
130
120
110
100
90
80
70
60
50
40
30
20
10
f1 (ppm)

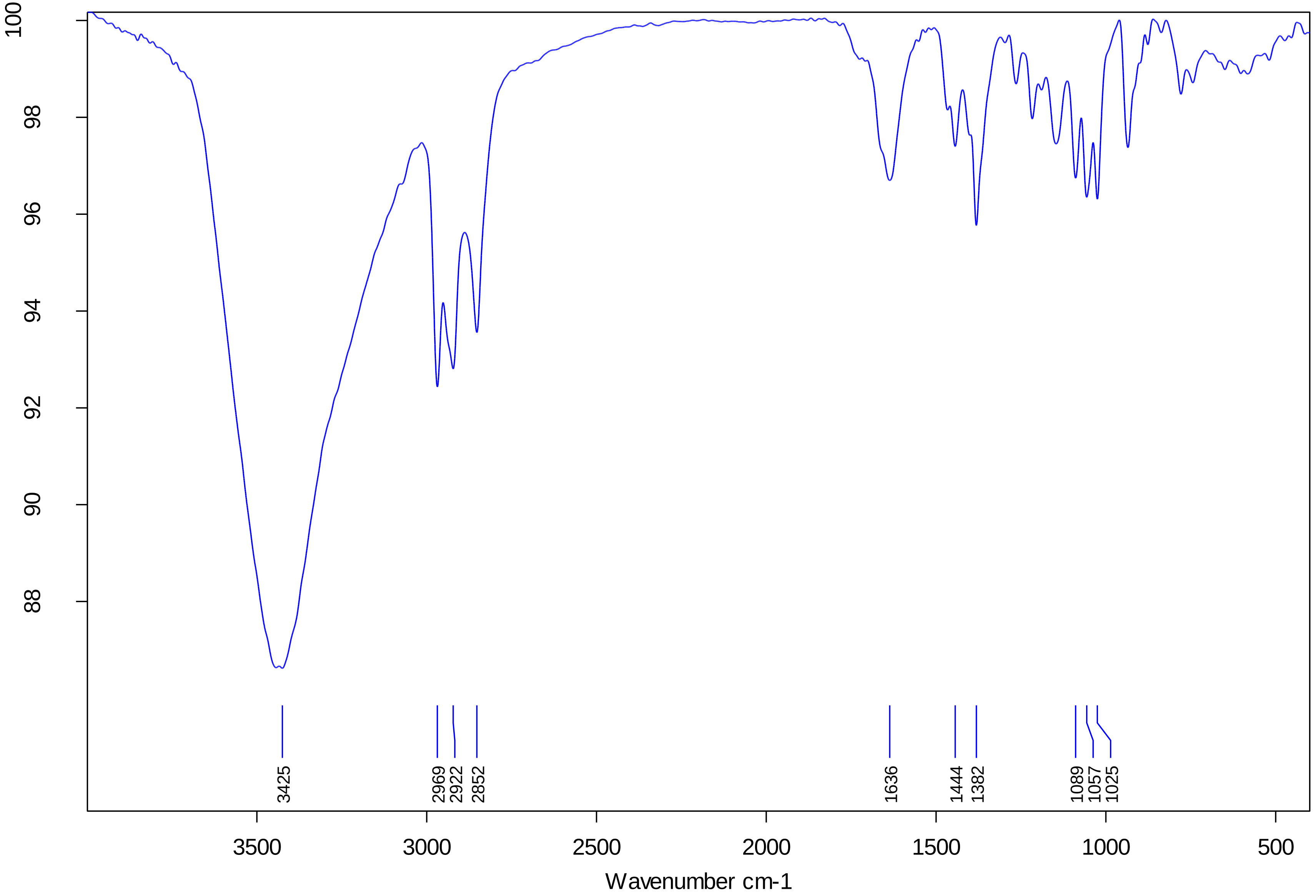

100
98
96
94
92
90
88
3425
2969
2922
2852
1636
1444
1382
1089
1057
1025
3500
3000
2500
2000
1500
1000
500
Wavenumber cm-1

^{1}H NMR, DEPT 135, ^{13}C NMR and IR of 2,6-dimethylocta-6,7-diene-2,5-diol (**7**)

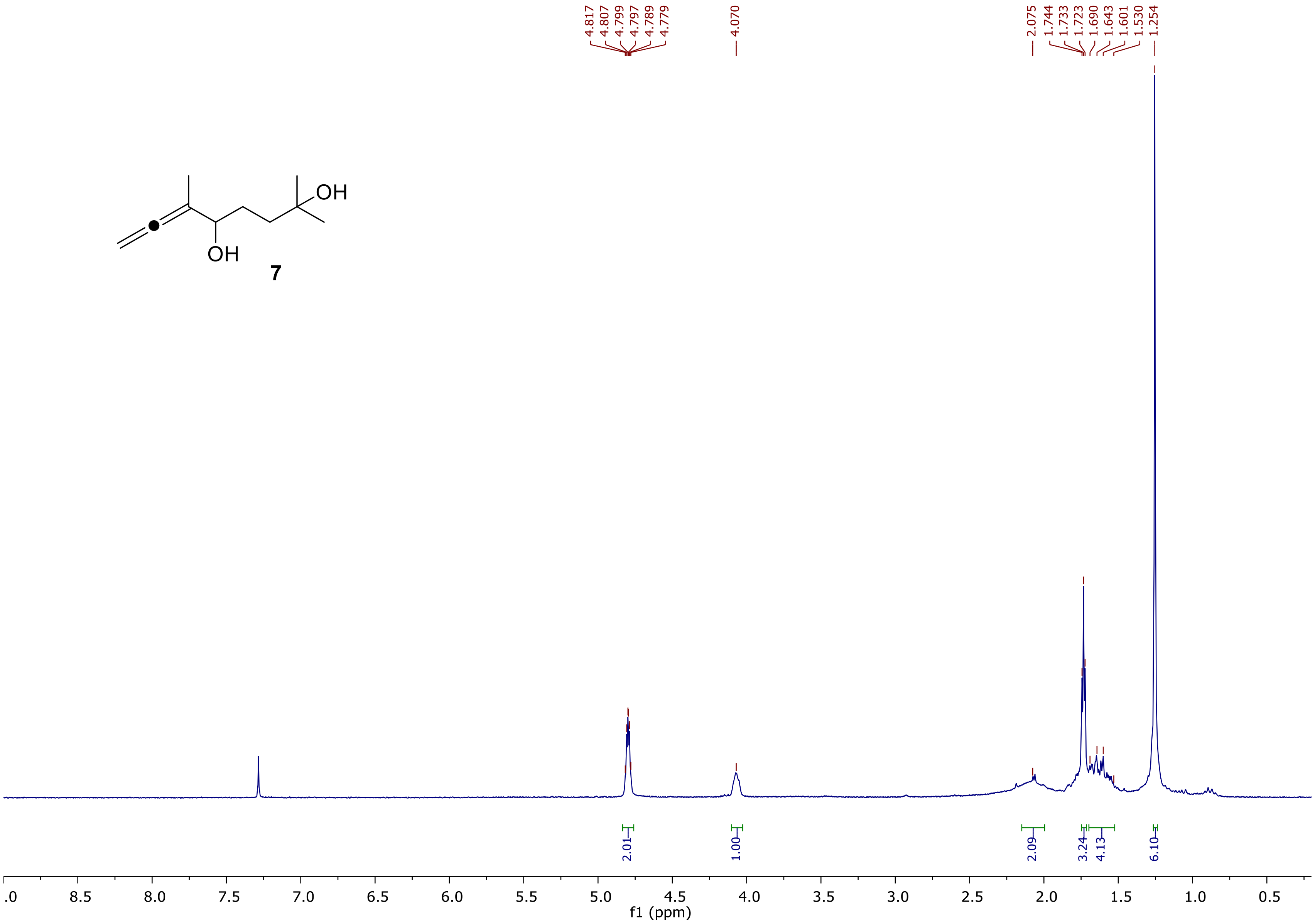

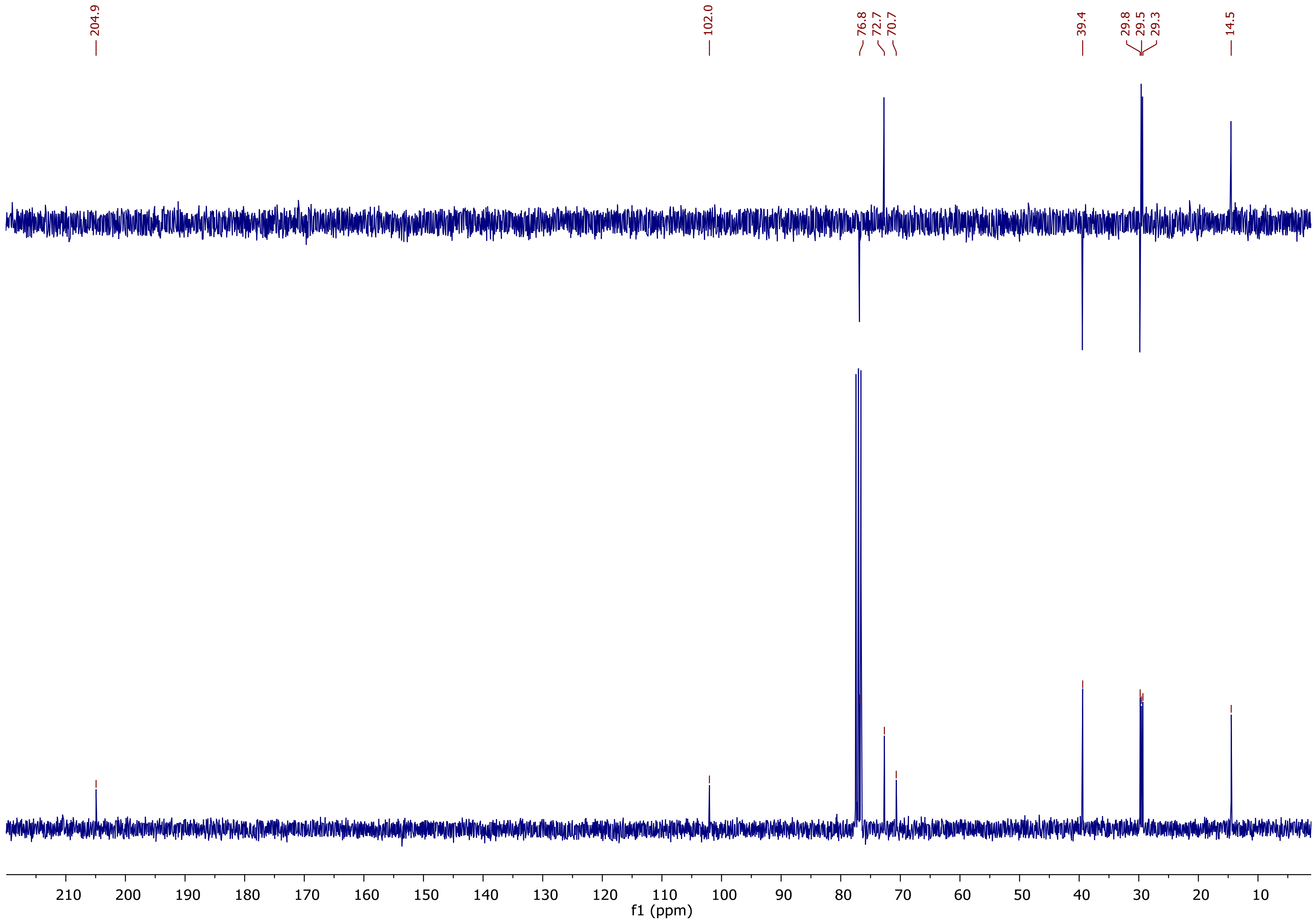

204.9
102.0
76.8
72.7
70.7
39.4
29.8
29.5
29.3
14.5
210 200 190 180 170 160 150 140 130 120 110 100 90 80 70 60 50 40 30 20 10
f1 (ppm)

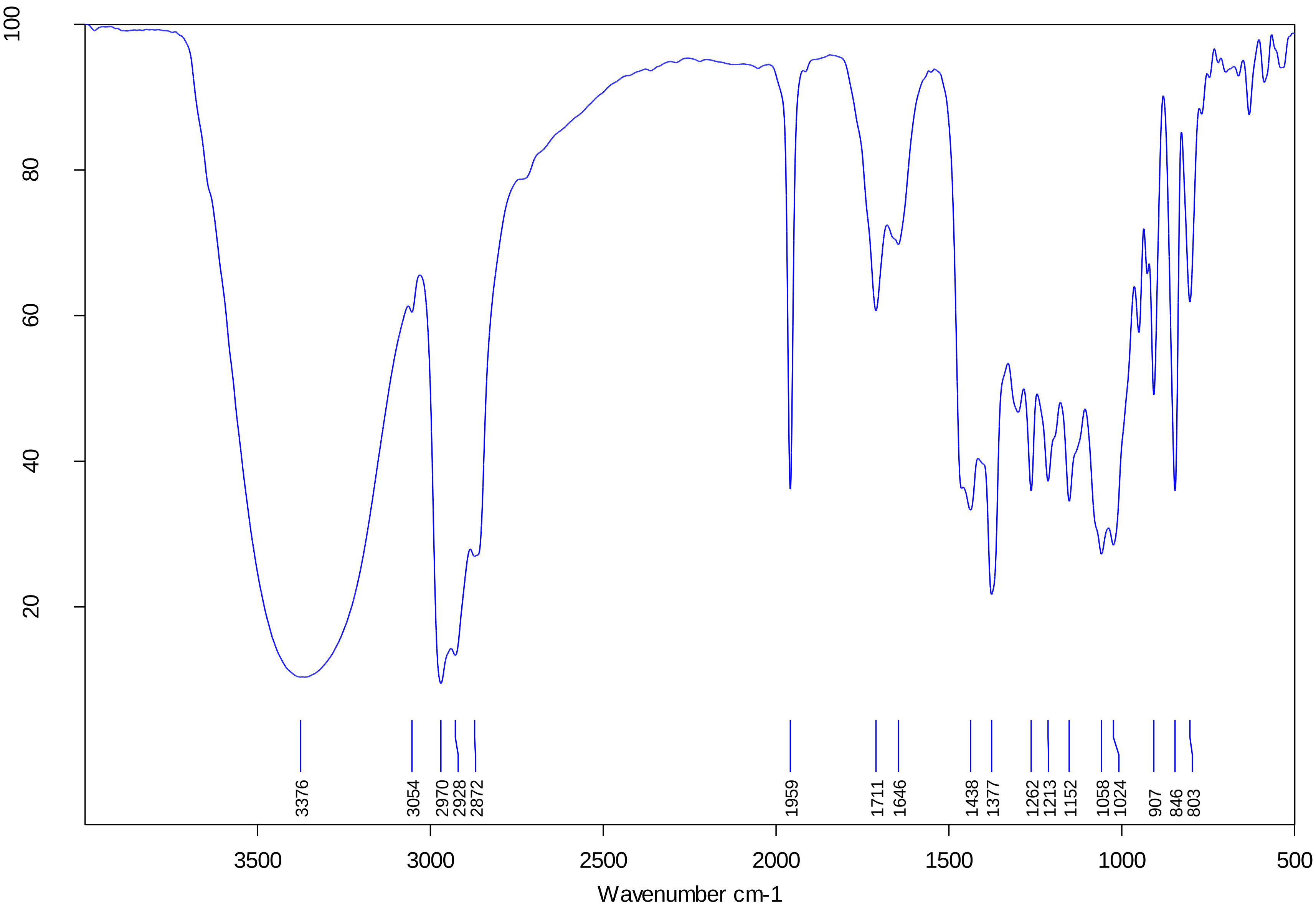
100
80
60
40
20
3376
3054
2970
2928
2872
1959
1711
1646
1438
1377
1262
1213
1152
1058
1024
907
846
803
3500
3000
2500
2000
1500
1000
500
Wavenumber cm-1

^{1}H NMR, DEPT 135 and ^{13}C NMR of ethyl (*S*)-5-methyl-4-(((*S*)-3,3,3-trifluoro-2-methoxy-2-phenylpropanoyl)oxy)hepta-5,6-dienoate ((*4S,2´S*)-**8**)

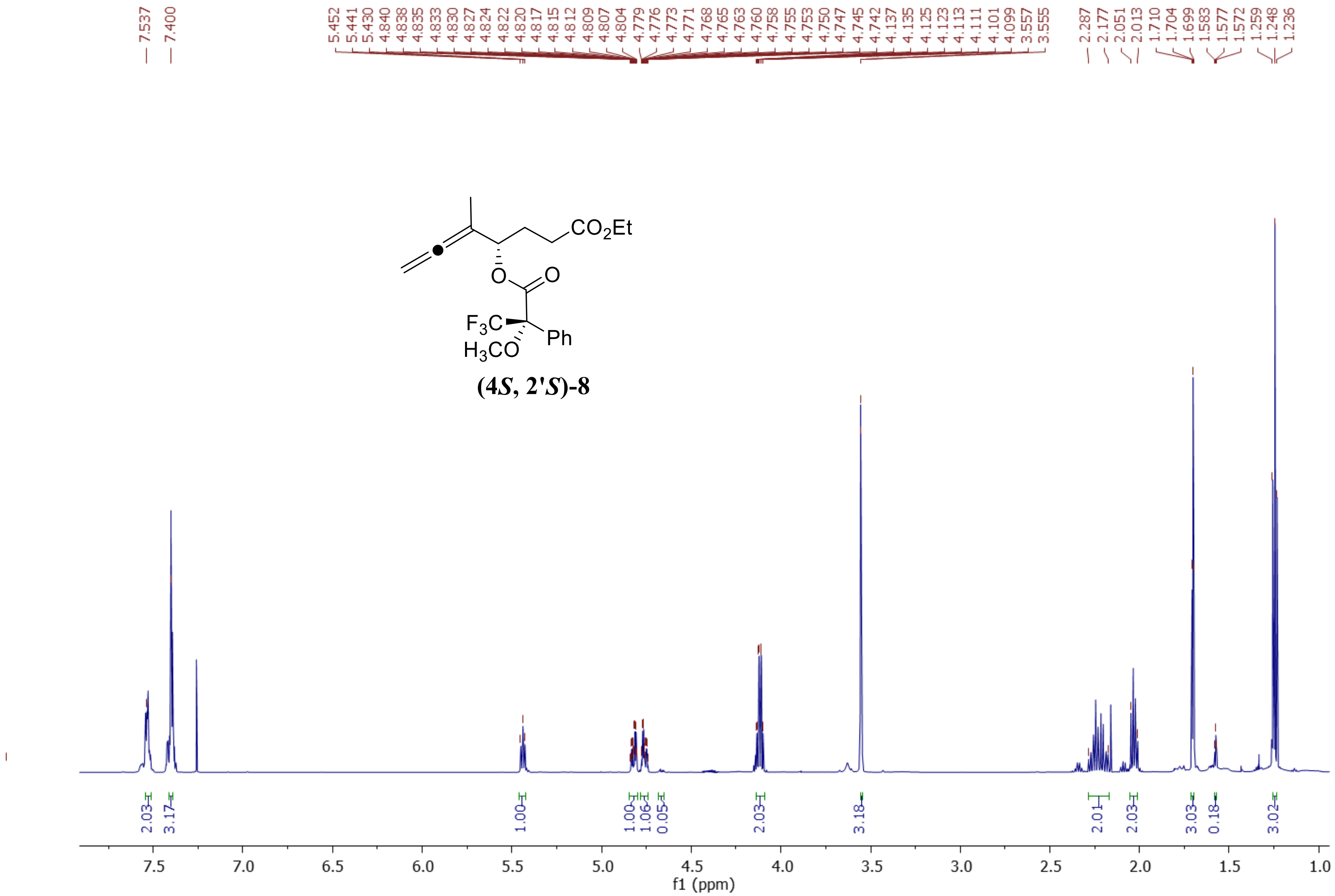

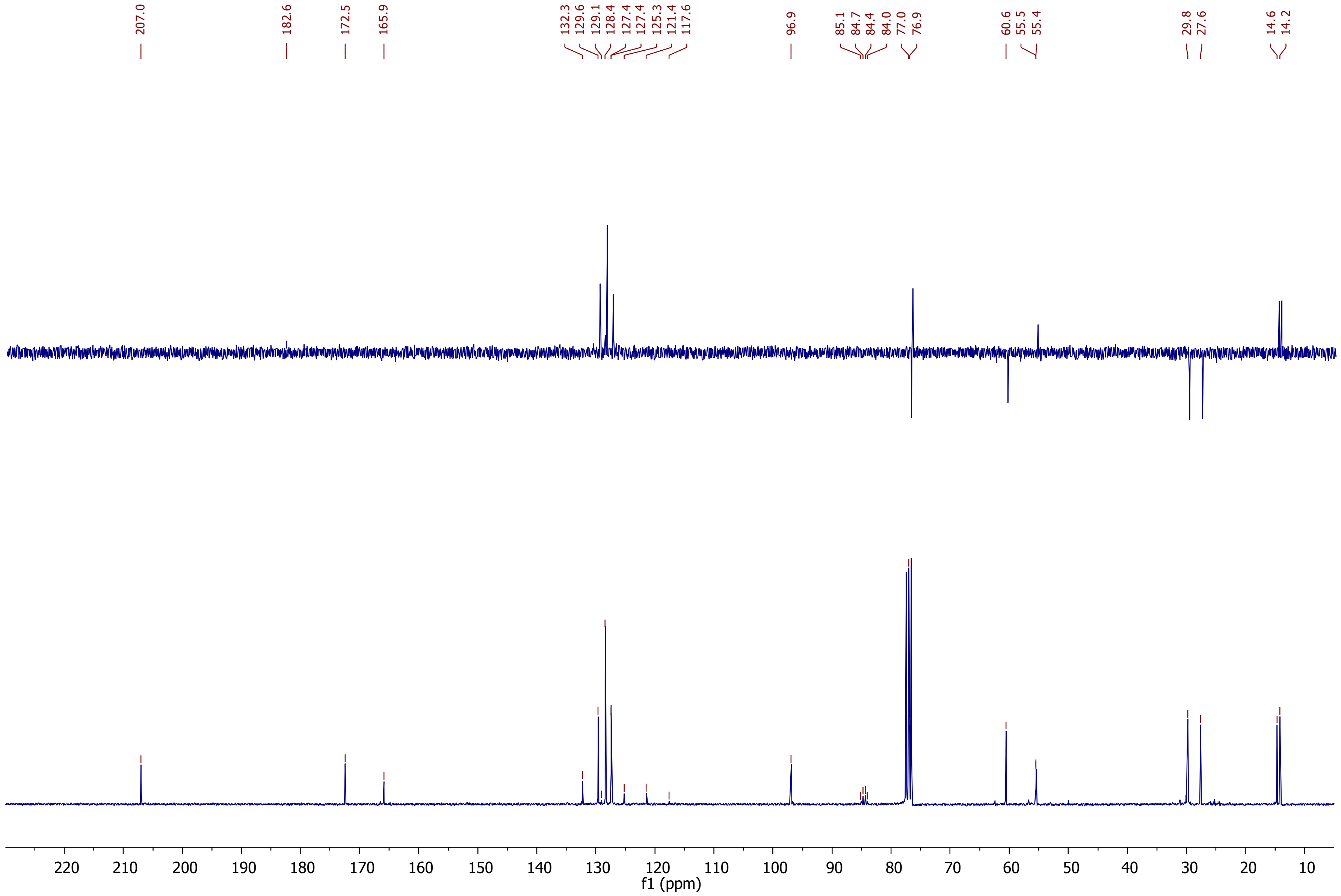

207.0
182.6
172.5
165.9
132.3
129.6
129.1
128.4
127.4
127.4
125.3
121.4
117.6
96.9
85.1
84.7
84.4
84.0
77.0
76.9
60.6
55.5
55.4
29.8
27.6
14.6
14.2
220 210 200 190 180 170 160 150 140 130 120 110 100 90 80 70 60 50 40 30 20 10
f1 (ppm)

^{1}H NMR, DEPT 135 and ^{13}C NMR of ethyl (*S*)-5-methyl-4-(((*R*)-3,3,3-trifluoro-2-methoxy-2-phenylpropanoyl)oxy)hepta-5,6-dienoate ((*4S,2´R*)-**8**)

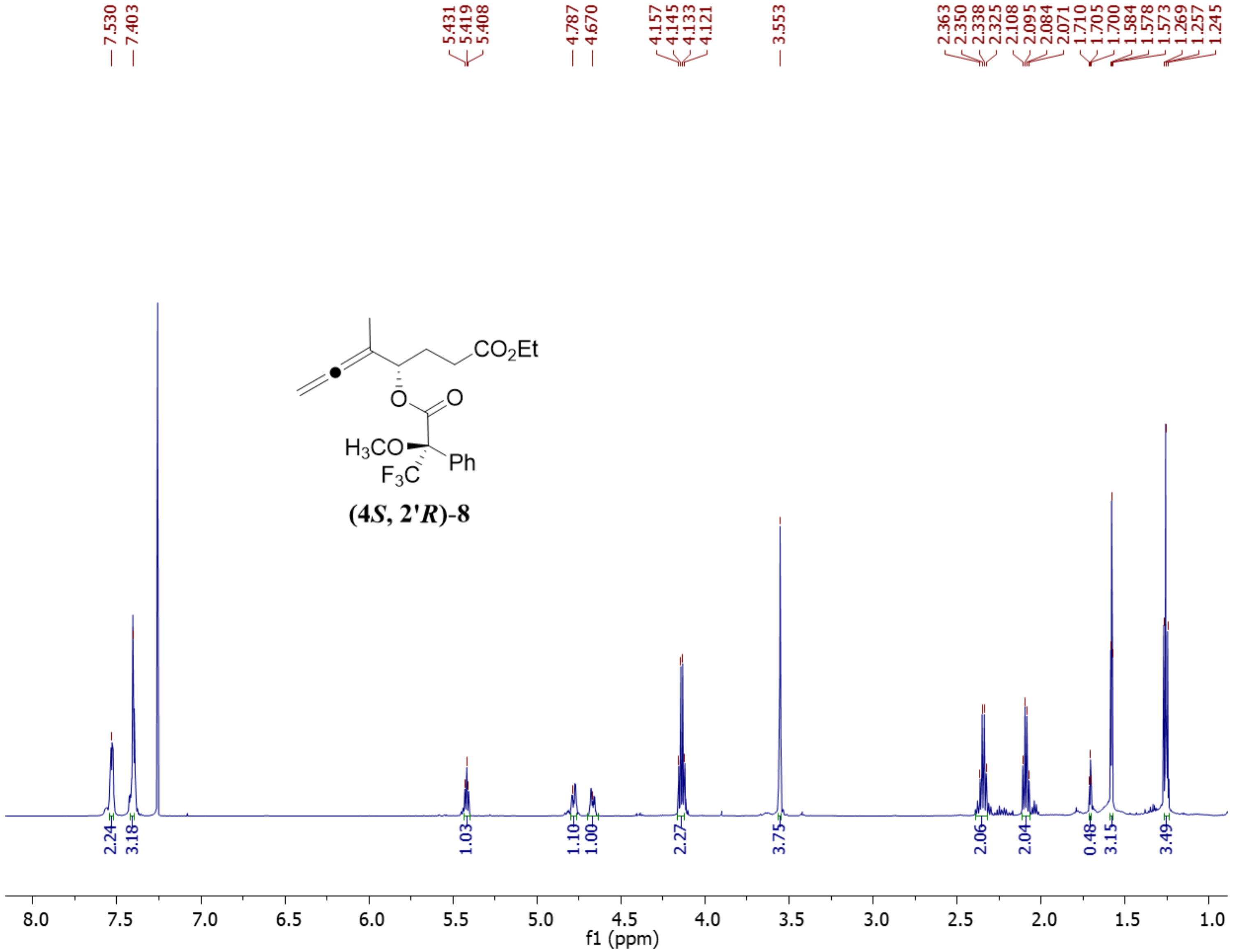

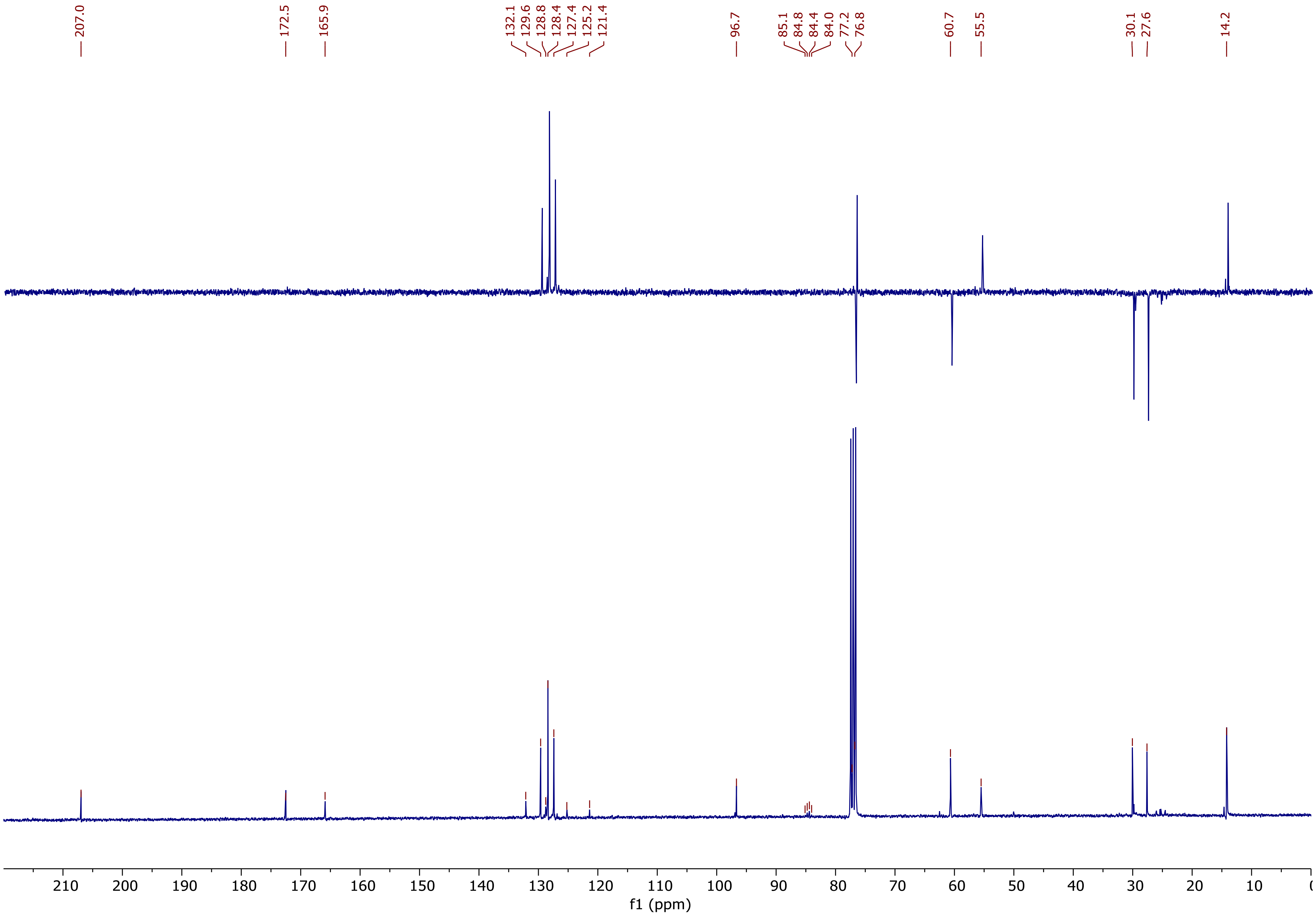
207.0
172.5
165.9
132.1
129.6
128.8
128.4
127.4
125.2
121.4
96.7
85.1
84.8
84.4
84.0
77.2
76.8
60.7
55.5
30.1
27.6
14.2
210 200 190 180 170 160 150 140 130 120 110 100 90 80 70 60 50 40 30 20 10 0
f1 (ppm)

^{1}H NMR analysis of diastereomeric MTPA esters

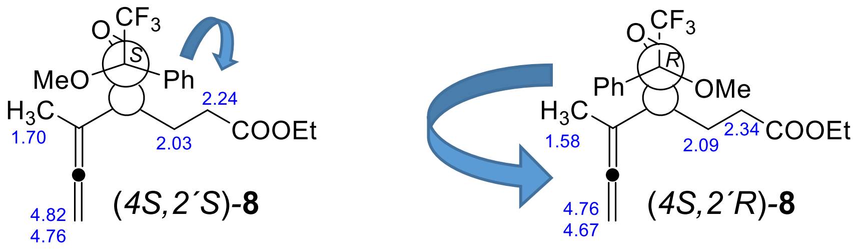

^{1}H NMR δ data (phenyl group shielding effect is indicated by the blue arrows in each representation)

^{19}F NMR: a) (*4S,2´S*)-8; b) (*4S,2´R*)-**8**

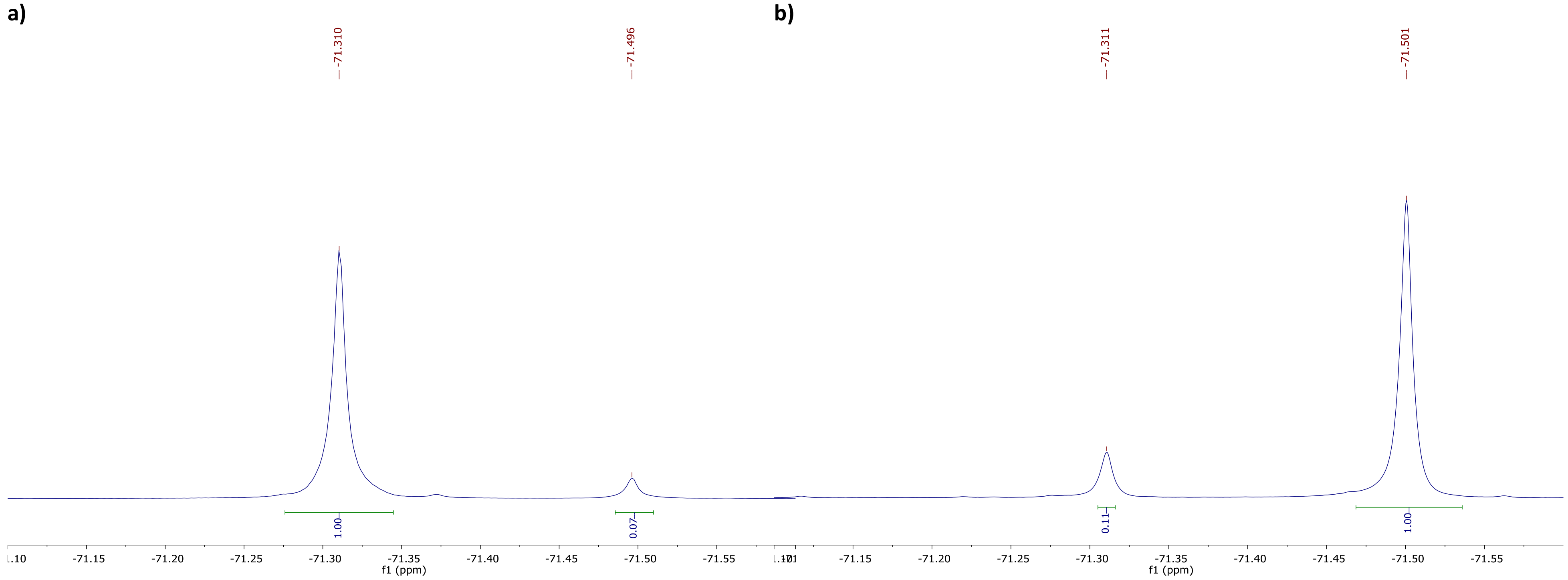

^{1}H NMR of diastereomeric mixture (*4S,2´S*)-**8** and (*4R,2´S*)-**8**

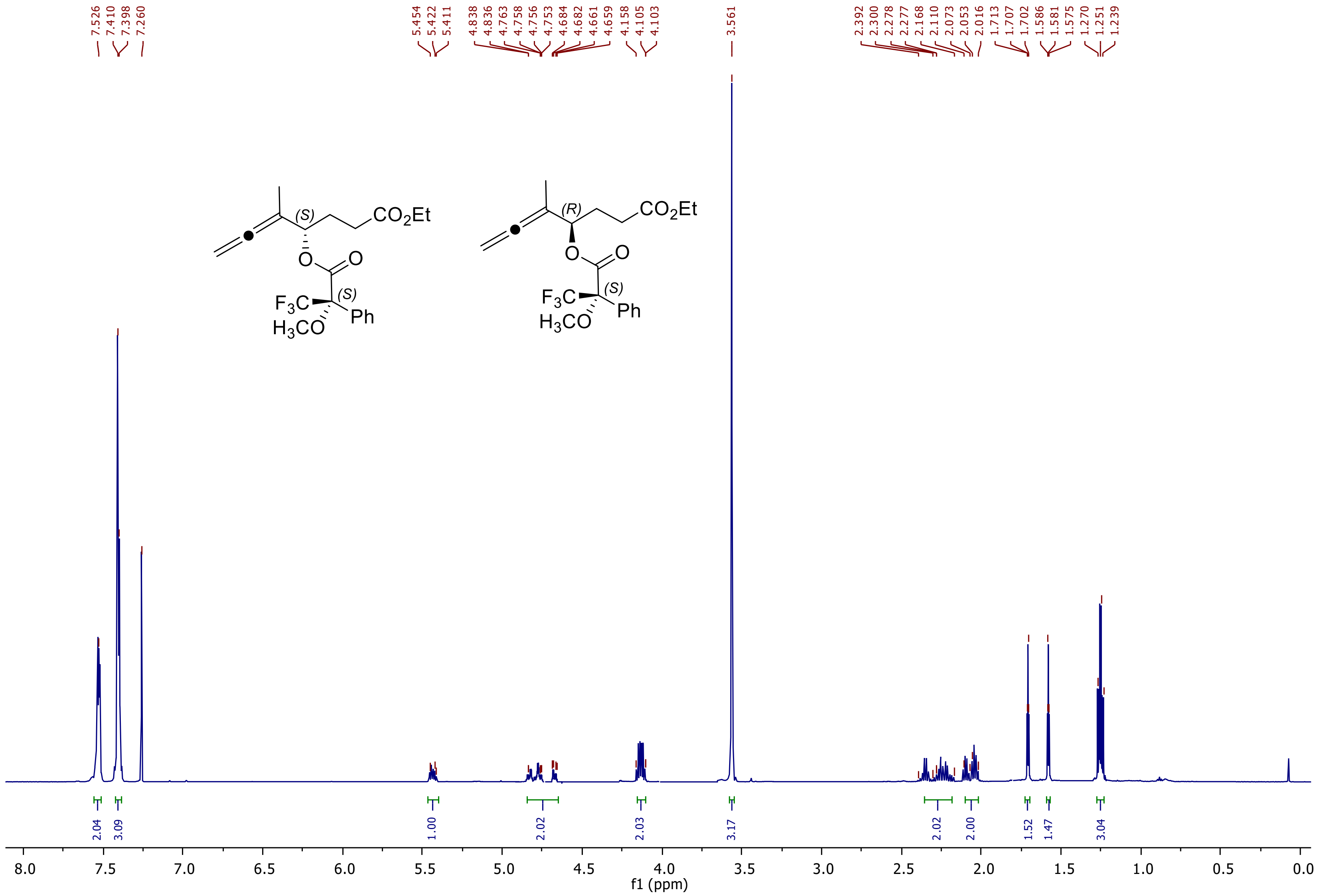

^{1}H NMR, DEPT 135, ^{13}C NMR and IR of (*R*)-ethyl 4-acetoxy-5-methylhepta-5,6-dienoate ((+)-**9**)

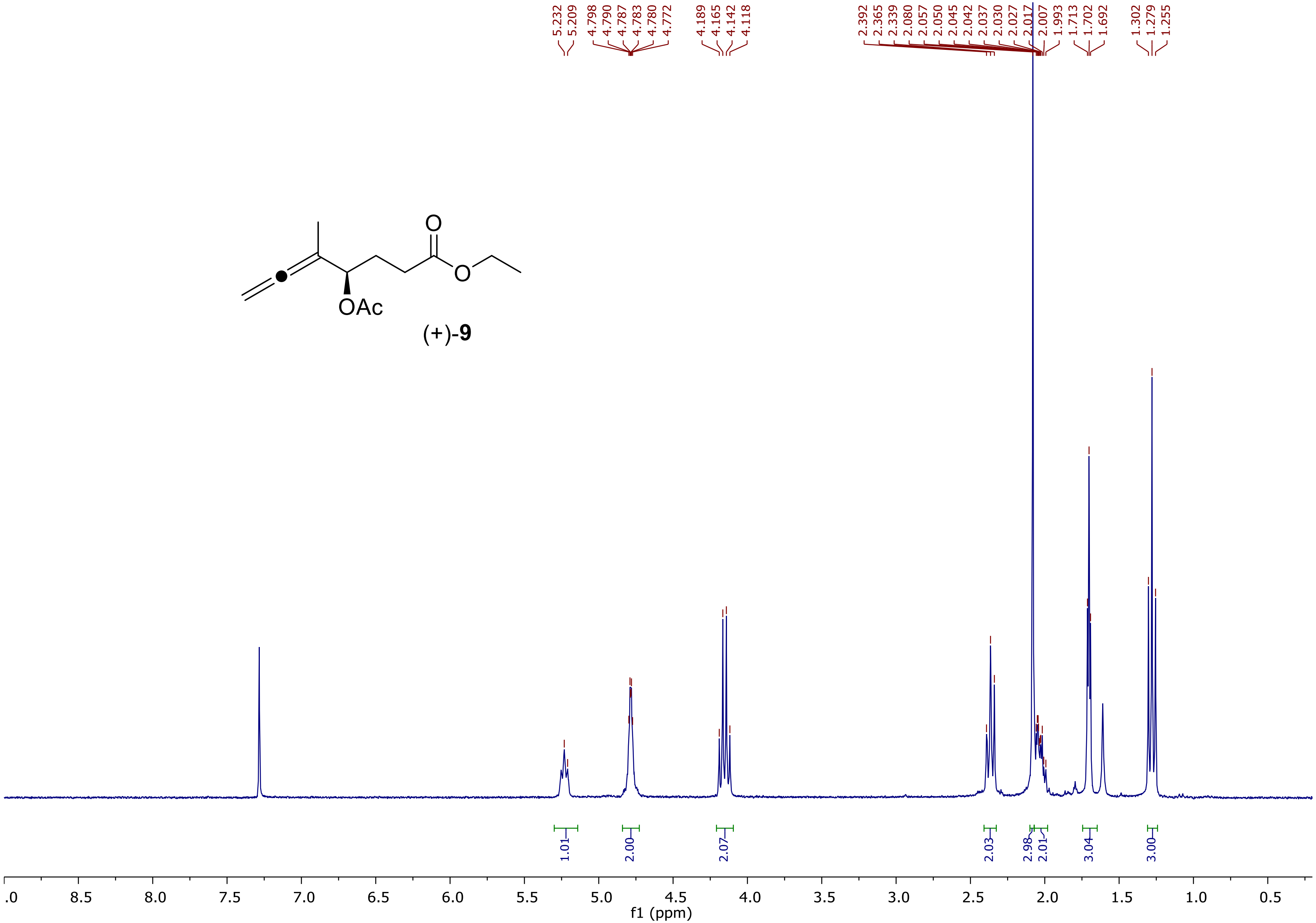

(+)-**9**

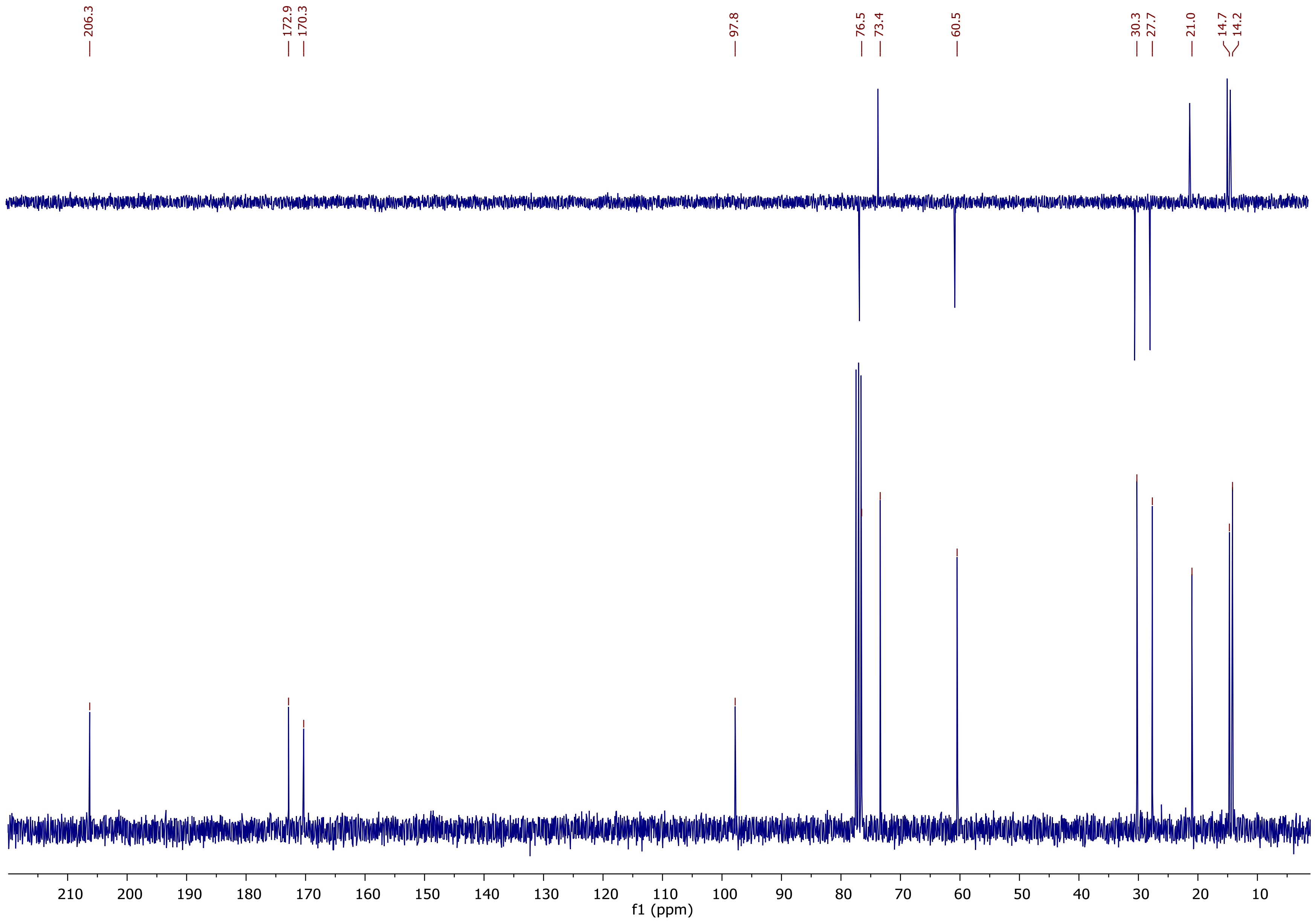
206.3
172.9
170.3
97.8
76.5
73.4
60.5
30.3
27.7
21.0
14.7
14.2
210 200 190 180 170 160 150 140 130 120 110 100 90 80 70 60 50 40 30 20 10
f1 (ppm)

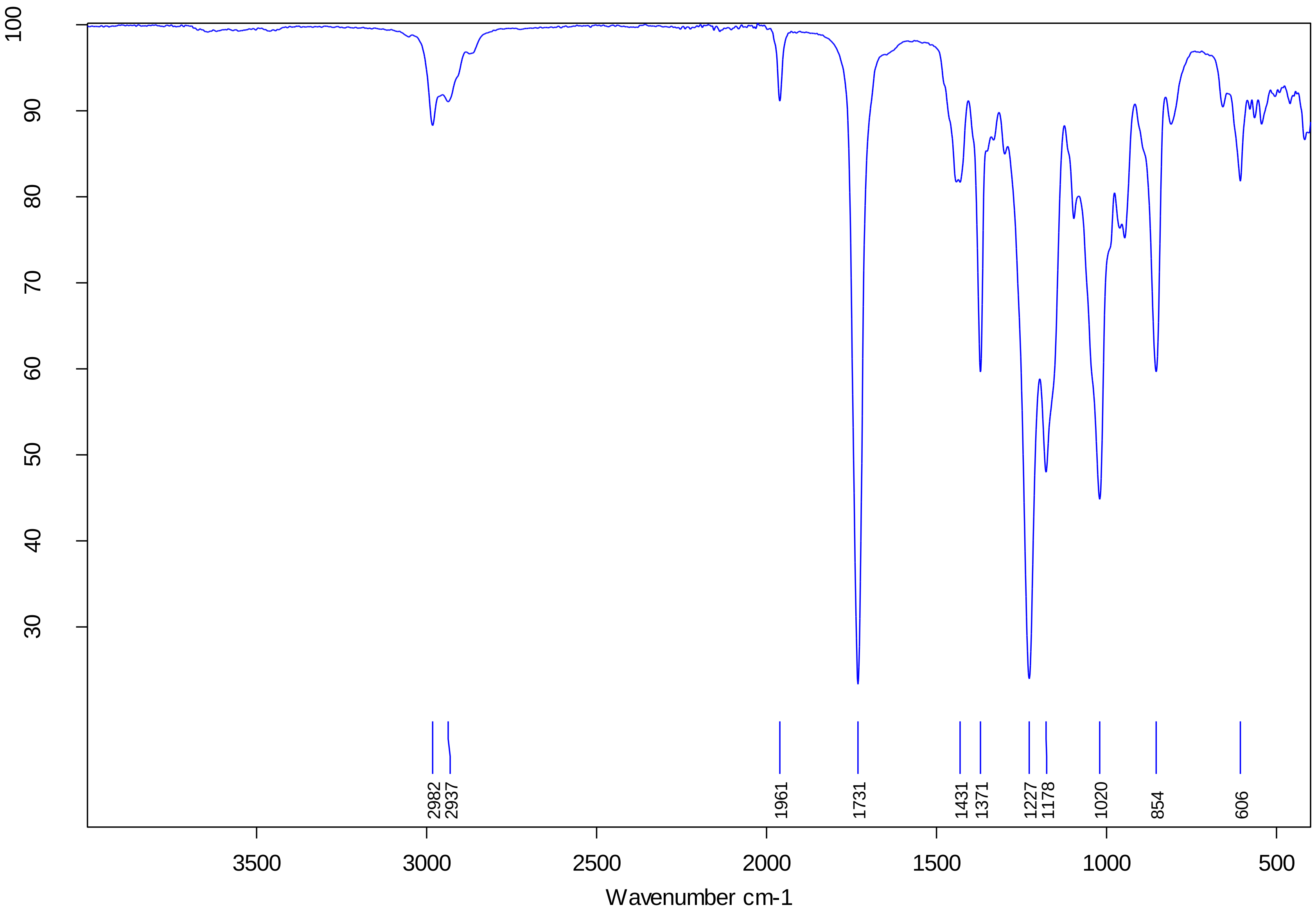
100
90
80
70
60
50
40
30
2982
2937
1961
1731
1431
1371
1227
1178
1020
854
606
3500
3000
2500
2000
1500
1000
500
Wavenumber cm-1

HPLC data

Ethyl 4-acetoxy-5-methylhepta-5,6-dienoate (*rac*-9)

System: Agilent 1100 series with a UV-DAD detector 210 nm. Column Daicel Chiracel OD-H

Solvent: hexane:iPrOH 99.5:0.5. Flow rate: 0.4 mL/min. Temperature: 25 °C

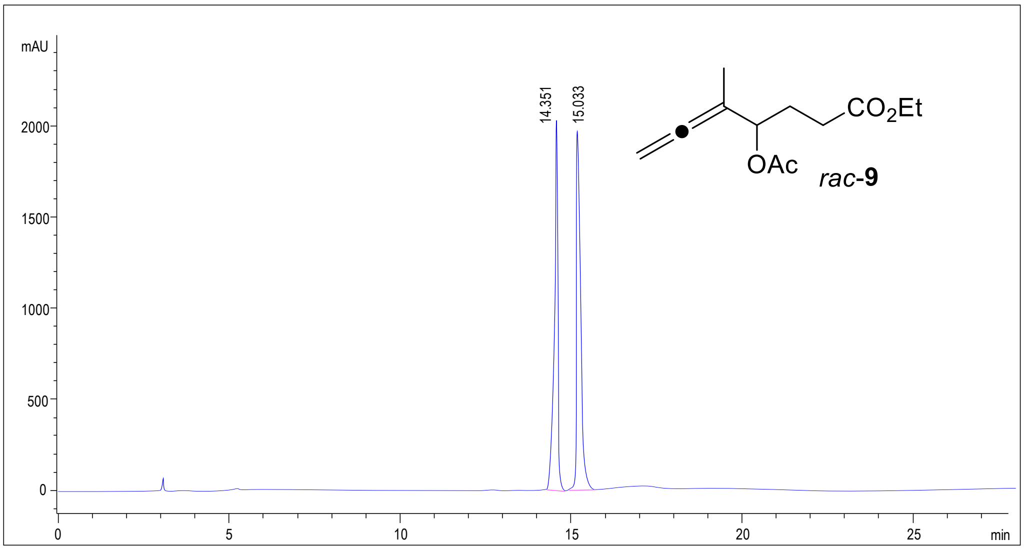

Peak #	RetTime [min]	Type	Width [min]	Area [mAU*s]	Height [mAU]	Area %
1	14.351	MM	0.1107	2.3657e4	2055.3609	50.0119
2	15.033	MM	0.1380	2.3645e4	1975.8990	49.9881
Totals:				47303.2237	4031.2599	

Ethyl (*R*)-4-acetoxy-5-methylhepta-5,6-dienoate ((+)-9)

System: Agilent 1100 series with a UV-DAD detector 210 nm. Column Daicel Chiracel OD-H

Solvent: hexane:iPrOH 99.5:0.5. Flow rate: 0.4 mL/min. Temperature: 25 °C

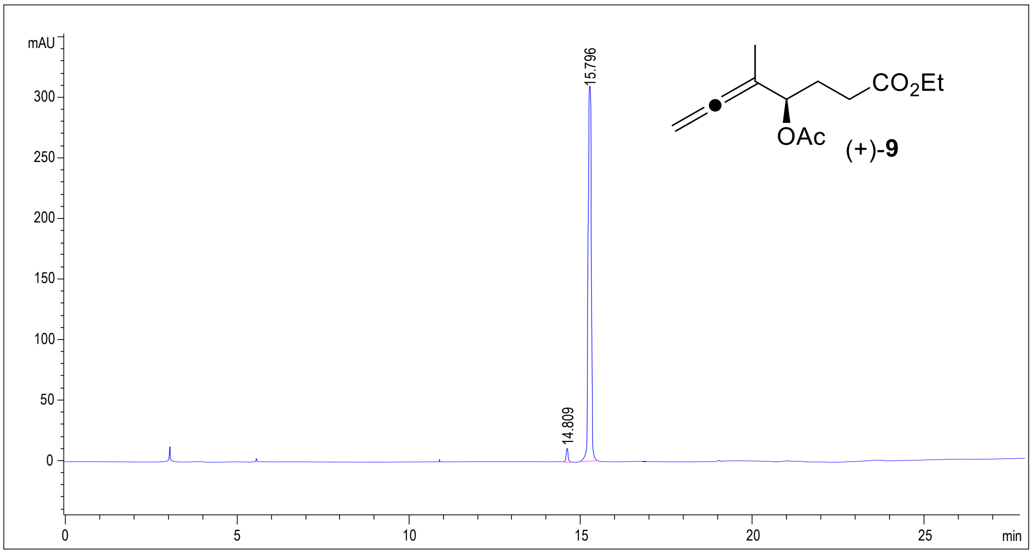

Peak #	RetTime [min]	Type	Width [min]	Area [mAU*s]	Height [mAU]	Area %
1	14.809	MM	0.1296	77.4528	11.9526	2.3525
2	15.796	MM	0.2075	3214.9666	309.8763	97.6475
Totals:				3292.4195	321.8289	

Ethyl 4-hydroxy-5-methylhepta-5,6-dienoate (*rac*-3)

System: Agilent 1100 series with a UV-DAD detector 210 nm. Column Daicel Chiralpack IA

Solvent: hexane:iPrOH 95:5. Flow rate: 0.5 mL/min. Temperature: 25 °C

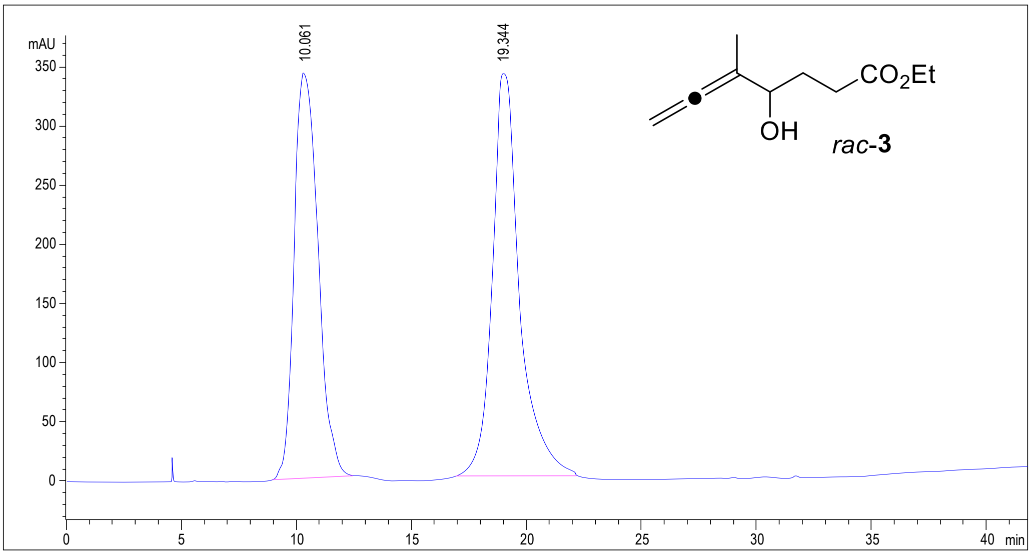

Peak #	RetTime [min]	Type	Width [min]	Area [mAU*s]	Height [mAU]	Area %
1	10.061	MM	1.7634	31319.8821	348.2564	48.9336
2	19.344	MM	1.8692	32684.9985	342.8645	51.0664
Totals:				64004.8806	691.1209	

Ethyl (*S*)-4-hydroxy-5-methylhepta-5,6-dienoate ((-)-3)

System: Agilent 1100 series with a UV-DAD detector 210 nm. Column Daicel Chiralpack IA

Solvent: hexane:iPrOH 95:5. Flow rate: 0.5 mL/min. Temperature: 25 °C

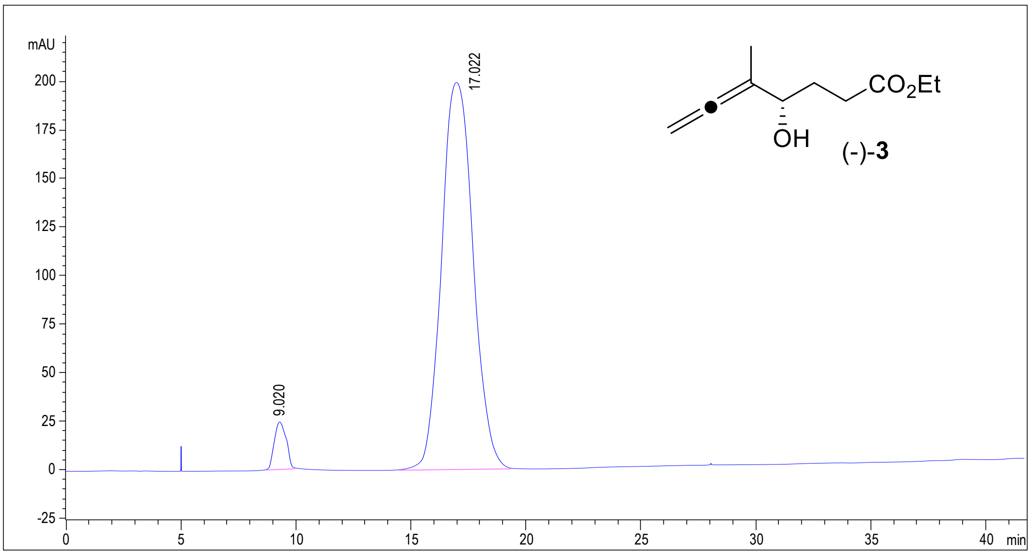

Peak #	RetTime [min]	Type	Width [min]	Area [mAU*s]	Height [mAU]	Area %
1	9.020	MM	0,8359	1072.1546	25.6527	5.0313
2	17.022	MM	2.0067	20237.5093	201.6994	94.9687
Totals:				21309.6639	227.3521	
